# Fibroblast-derived CCL2 orchestrates immune responses and defends against *Staphylococcus aureus* skin infection

**DOI:** 10.1038/s41423-026-01442-7

**Published:** 2026-06-22

**Authors:** Tatsuya Dokoshi, Marta Palomo-Irigoyen, Kazuki Dai, Terumi Hashimoto, Hiroaki Konishi, Lauriane Hivert, Michelle Bagood, Hung Chan, Samia Almoughrabie, Yoshiyuki Nakamura, Kellen J. Cavagnero, Teruaki Nakatsuji, Mikihiro Fujiya, Richard L. Gallo

**Affiliations:** 1 Department of Dermatology, University of California, La Jolla, CA USA; 2 Department of Internal Medicine, Division of Gastroenterology, Asahikawa Medical University, Asahikawa, Japan; 3 Genes and Disease Laboratory, Department of Dermatology, Medical University of Vienna, Vienna, Austria; 4 Department of Dermatology, University of Tsukuba: Tsukuba, Ibaraki, Japan

**Keywords:** Fibroblast, CCL2, Staphylococcus aureus, Skin infection, Spatial transcriptomics, Innate immunity, Bacterial infection, Antimicrobial responses

## Abstract

Host defense against invasive bacterial infections of the skin is essential for survival. It involves a complex yet incompletely understood process of microbial recognition followed by innate and adaptive systems for communication between resident and recruited cells to mount an effective defense. Stromal fibroblasts have not been classically considered immunocytes but are gaining recognition for their critical role in inflammation. Here, we identify fibroblast-derived C-C motif chemokine ligand 2 (CCL2) as a key mediator of host defense against invasive *Staphylococcus aureus* infection. Single-cell RNA sequencing revealed that fibroblasts are a predominant source of CCL2 under steady-state conditions in both human and mouse skin. The use of mice with a conditional deletion of CCL2 in fibroblasts demonstrated that the expression of CCL2 by fibroblasts alters macrophage cytokine production and antigen-presentation-associated responses and is important for monocyte recruitment. Additionally, we revealed a novel role for fibroblast-derived CCL2 in promoting fibroblast-to-adipocyte differentiation via ERK and p38 signaling, leading to reactive adipogenesis and enhanced production of the antimicrobial peptide cathelicidin. In mice with targeted deletion of *Ccl2* in fibroblasts, these host immune responses are impaired, and *S. aureus* infection of the skin is greatly increased. These findings highlight fibroblast-derived CCL2 as a critical regulator of immunity and suggest its broader implications in inflammatory and infectious diseases.

## Introduction

Recent studies have highlighted the significant role of transcriptionally distinct fibroblast subsets in regulating immune responses in barrier tissues by producing antimicrobial molecules, recruiting immune cells, and organizing the extracellular matrix [1–4]. However, despite these emerging insights, the precise mechanisms by which fibroblasts regulate immune responses, especially during infection, remain incompletely understood [5]. In particular, the interactions between fibroblasts and macrophages in the context of infection are not well characterized.

Fibroblasts express and produce a variety of cytokines and chemokines, such as IL-1, IL-6, CCL2, and CCL7, in response to inflammation, indicating strong interactions between fibroblasts and immune cells [6–9]. Previous research has demonstrated that dermal fibroblasts in the preadipocyte lineage act to resist *Staphylococcus aureus* (*S. aureus*) infection in the skin through the production of cathelicidin antimicrobial peptide (CAMP) [2]. More recently, fibroblasts were shown to be required for normal neutrophil recruitment in response to IL-17 and TNFα, which is also an essential innate immune response to infection [9]. Additionally, while fibroblast‒macrophage interactions have been explored in the contexts of tissue repair, fibrosis, and tumor microenvironments [10–12], the mechanism through which fibroblasts influence macrophage functions during infection is unclear. In this study, we sought to identify critical communication events that enable fibroblasts to contribute to host defense against *S. aureus* infection in the skin.

An important chemokine for the control of infection is CCL2, also known as MCP-1, which recruits CCR2+ monocytes/macrophages [13, 14]. CCL2 is produced by various cell types during inflammation and has been shown to influence monocyte recruitment, polarization, and macrophage activation, promoting phagocytosis and the production of interleukins such as IL-1b and IL-10 [15, 16]. In the bone marrow, CCL2 retains monocytes, whereas in epithelial tissues, it activates monocytes/macrophages, enhancing immune responses such as phagocytosis and cytokine expression. These events are relevant in vivo, as the administration of recombinant CCL2 or bone marrow-derived CCL2+ mesenchymal stromal cells (MSCs) has been shown to improve wound healing in the skin and colon [17, 18]. While epithelial-derived CCL2 has been implicated in wound healing through the modulation of IL-10 production by macrophages, the role of fibroblast-derived CCL2 in infection and inflammation remains unexplored.

In this study, we investigated the role of fibroblast-derived chemokines in the defense against *S. aureus* infection. We found that fibroblasts predominantly express CCL2 in both human and mouse single-cell RNA sequencing (scRNA-seq) datasets under steady-state conditions. Using a fibroblast-specific Ccl2 knockout mouse model, we showed that these mice exhibit increased susceptibility to *S. aureus* infection. scRNA-seq and spatial sequencing revealed that increased susceptibility to infection is associated with the combined activity of CCL2 to modulate macrophage function and promote the differentiation of fibroblasts into mature adipocytes via activation of the ERK and p38 pathways. Taken together, our findings reveal how fibroblasts in the dermis play a critical role in the defense against *S. aureus* infection through their ability to express CCL2.

## Results

### Fibroblasts express CCL2, CXCL12, and IL6

**Fig. 1:**
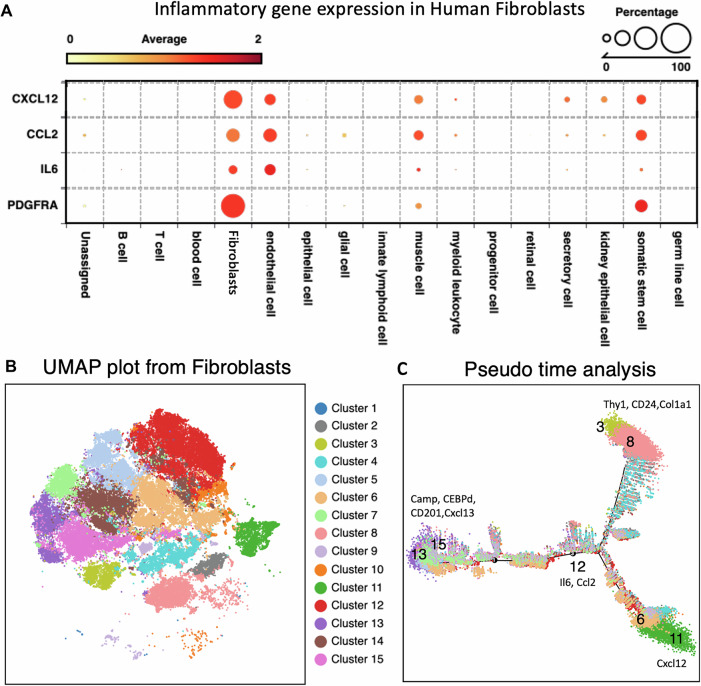
Human fibroblasts express high levels of CCL2, CXCL12, and IL6. Human fibroblasts were extracted from a published single-cell RNA-sequencing dataset (GSE201333) and reanalyzed. **A** Expression of inflammatory genes across major cell types in the human dataset. **B** UMAP visualization of extracted fibroblasts. **C** Pseudotime analysis of fibroblast clusters and distribution of selected inflammatory genes

**Fig. 2:**
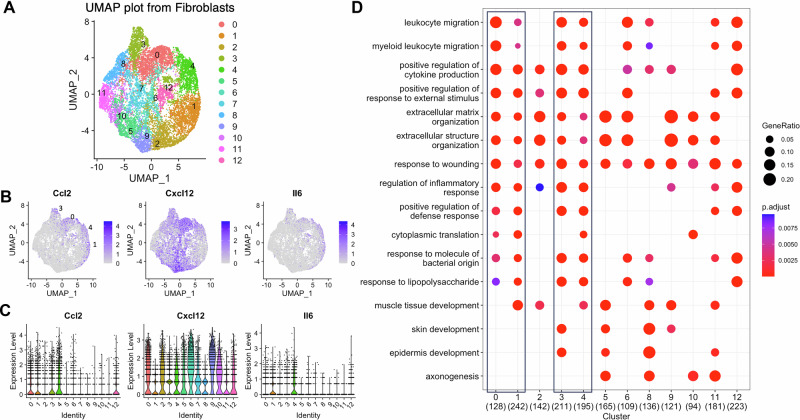
Mouse skin and colon fibroblasts contain distinct subsets of Ccl2-, Cxcl12-, and Il6-expressing cells. Mouse fibroblasts were extracted from published single-cell RNA-sequencing datasets and reanalyzed. **A** UMAP visualization of extracted fibroblasts. **B** Feature plots showing the expression of Ccl2, Cxcl12, and Il6. **C** Violin plots showing the expression of Ccl2, Cxcl12, and Il6 across fibroblast clusters. **D** Top Gene Ontology (GO) terms associated with each fibroblast cluster. Skin and colon fibroblasts were analyzed separately, as shown in the corresponding supplementary panels

To identify cytokines and chemokines expressed by fibroblasts, we first analyzed a large single-cell RNA sequencing (scRNA-seq) dataset from a multiorgan human tissue atlas [19] across cell types. This analysis revealed that compared with other cell types, fibroblasts express more CCL2, CXCL12 and IL6 (Fig. 1A and Supplementary Fig. 1a). Further analysis of the fibroblast populations distinguished by Pdgfra expression revealed 15 different clusters in the UMAP plot (Fig. 1B). Pseudotime analysis revealed that cluster 12 (CCL2 and IL6 high) and cluster 11 (CXCL12 high) were distinct from clusters 3 and 8 (THY1- and CD24-high) and clusters 13 and 15 (CEBPD- and CAMP-high) (Fig. 1C). Analysis of independent datasets of mouse skin and colon [20, 21] also revealed clusters of fibroblasts expressing Ccl2 and Il6 (clusters 0, 1, 3, and 4), which were distinct from clusters expressing Cxcl12 (clusters 6 and 9) (Fig. 2A–C and Supplementary Fig. 1b). GO term enrichment analysis revealed that the Ccl2- and Il6-high clusters were associated with the regulation of the inflammatory response in both skin and colon tissue (Fig. 2D and Supplementary Fig. 2a–c).

### Fibroblast-derived CCL2 recruits macrophages and alters immune functions

**Fig. 3:**
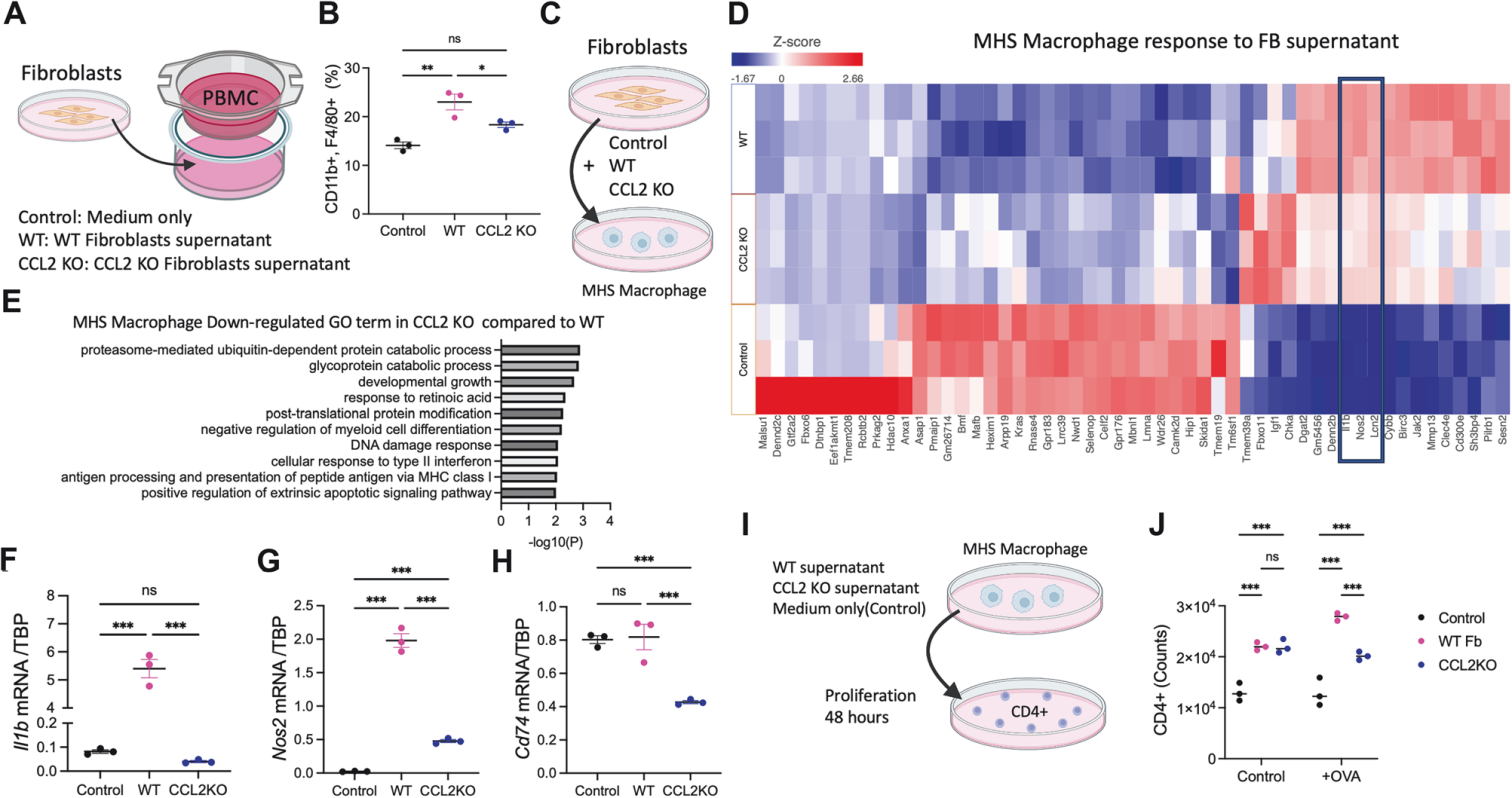
Fibroblast-derived CCL2 promotes monocyte chemotaxis and modulates macrophage immune responses. **A** Schematic of the chemotaxis assay. Mouse peripheral blood mononuclear cells (PBMCs) were placed in the upper chamber, and fibroblast-conditioned media were placed in the lower chamber. Control, unconditioned medium; WT, conditioned medium from wild-type fibroblasts; KO, conditioned medium from *Ccl2*-deficient fibroblasts. The number of migrated cells was quantified after 3 h by flow cytometry. **B** Ratio of migrated monocyte/macrophage populations in the lower chamber. **C** Schematic of the macrophage-conditioning assay. Mouse MHS macrophages were cultured for 24 h in control, WT, or KO medium as defined above. **D** Heatmap of bulk RNA-seq data from MHS macrophages under the indicated conditions. **E** Top downregulated GO terms in macrophages treated with conditioned medium from KO fibroblasts compared with those treated with conditioned medium from WT fibroblasts. **F–H** qPCR analysis of Il1b, Nos2, and Cd74 expression in MHS macrophages. **I** Schematic of the T-cell proliferation assay using conditioned MHS macrophages. **J** CD4 + T-cell number after 48 h of coculture. Statistical significance was determined using ordinary one-way ANOVA with Tukey’s multiple-comparison test or ordinary two-way ANOVA with Sidak’s multiple-comparison test, as indicated. The error bars represent the mean ± SEM. *P < 0.05, **P < 0.01, ***P < 0.001. Each experiment was repeated at least three times

CCL2 is a potent chemokine that recruits monocytes, T cells, B cells, natural killer cells, basophils, dendritic cells, myeloid-derived suppressor cells, and neutrophils while also influencing macrophage development; therefore, it is a prime candidate to further explore as a key mediator of host defense from fibroblasts [16, 22, 23]. To determine whether the CCL2 produced by fibroblasts contributes to promoting chemotactic activity, we tested whether fibroblasts can recruit monocytes from peripheral blood mononuclear cells (PBMC) (Fig. 3A). Culture medium from mouse primary fibroblasts promoted the recruitment of neutrophils and monocytes, whereas culture medium from *Ccl2*-deficient fibroblasts reduced monocyte/macrophage chemotactic activity (Fig. 3B and Supplementary Fig. 3a–d).

Next, to determine whether fibroblast-derived Ccl2 alters macrophage responses, we cultured MHS macrophages with fibroblast-conditioned medium (Fig. 3C). Bulk transcriptome sequencing analysis revealed that culture medium conditioned by wild-type primary dermal fibroblasts altered macrophage gene expression. Compared with those in the unconditioned control medium, the transcriptomic profiles of the macrophages in the conditioned media from the wild-type and *Ccl2*-deficient fibroblast-conditioned media were similar overall, but compared with the control medium, the *Ccl2*-deficient condition resulted in lower expression of multiple immune-related genes, including Il1b, Nos2, and Cd74 (Fig. 3D and Supplementary Fig. 3e). Gene Ontology analysis revealed differential enrichment of several GO terms, including negative regulation of myeloid cell differentiation, in MHS cells exposed to wild-type fibroblast-conditioned media compared with *Ccl2*-deficient fibroblast-conditioned media (Fig. 3E). qPCR confirmed a decrease in the expression of immune genes, including Il1b, Nos2, and CD74, in MHS cells exposed to conditioned media from *Ccl2*-deficient fibroblasts or a CCL2 inhibitor (Fig. 3F–H and Supplementary Fig. 3f). Conditioned culture media from MHS cells activated by conditioned media from wild-type or *Ccl2*-deficient fibroblasts induced T-cell proliferation (Fig. 3I, J). Furthermore, ovalbumin (OVA)-stimulated macrophages enhanced T-cell proliferation in a CCL2-dependent manner.

Taken together, these findings indicate that Ccl2 produced by cultured fibroblasts contributes to monocyte recruitment and modulates macrophage immune gene expression in this in vitro setting.

### Defenses of fibroblast-derived CCL2 against *S. aureus*

**Fig. 4:**
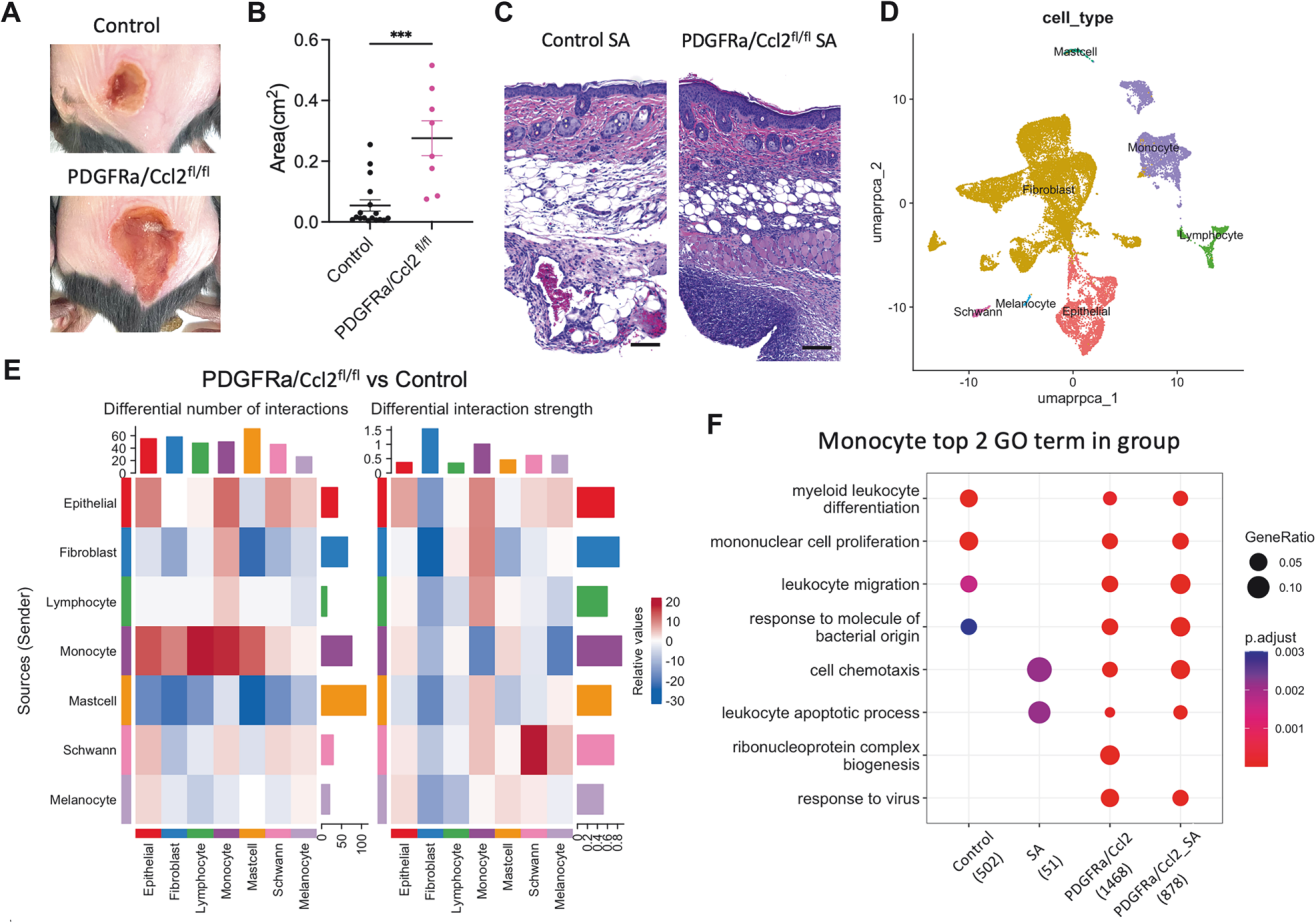
Fibroblast-derived CCL2 contributes to host defense against *S. aureus* infection. Control and Pdgfra/Ccl2^fl/fl^ mice were intradermally injected with *S. aureus* USA 300 (MRSA, 5 × 10^5^ CFU) on the back skin. **A** Representative macroscopic image of infected skin. **B** Lesion size in the infection area after 3 days of infection. **C** Hematoxylin and eosin (H&E) staining of infected skin on day 3. **D** UMAP plot of single-cell sequencing data from control, Pdgfra/Ccl2^fl/fl^, *S. aureus*-infected, and Pdgfra/Ccl2^fl/fl^ and *S. aureus*-infected mice. **E** Results from the CellChat analysis showing relative changes in the number and strength of cell‒cell interaction signals in PDGRRa/Ccl2^fl/fl mice^ compared with those in control mice. **F** Top 2 Gene Ontology (GO) terms identified in monocyte clusters from each experimental group. Scale bar: 50 microns. Statistical significance was determined using Student’s unpaired two-sided t test (**B**). Error bars indicate the mean ± SEM; * *P* < 0.05, ** *P* < 0.01, *** *P* < 0.001. Each experiment was repeated at least three times

To test the role of fibroblast-derived Ccl2 in host defense against infection in vivo, we generated fibroblast-specific Ccl2 knockout mice by crossing Pdgfra-Cre mice with Ccl2^fl/fl^ mice (Pdgfra/Ccl2^fl/fl^). These mice, along with their Cre-negative control littermates, were challenged with an intradermal injection of *Staphylococcus aureus* USA300 (*S. aureus*). Mice deficient in Ccl2 in fibroblasts exhibited increased susceptibility to *S. aureus* infection, as reflected by increased lesion area (Fig. 4A, B), heightened perilesional tissue inflammation (Fig. 4C) and greater bacterial survival, as estimated by IVIS imaging of bacterial luminescence (Supplementary Fig. 4a, b). scRNA-seq analysis of these skin lesions revealed monocyte-associated clusters, and condition-specific differences in cell-type distribution were further assessed by separate UMAP visualizations and relative abundance analyses (Fig. 4D and Supplementary Fig. 4c–e). Cell‒cell interaction analysis using CellChat revealed stronger interactions between monocytes and other cell types and fewer fibroblast autocrine interactions in Pdgfra/Ccl2^fl/fl^ mice (Fig. 4E). More specifically, immune cell signaling, such as MHC-II, CD80, and CD86 signaling, was downregulated in monocytes from Pdgfra/Ccl2^fl/fl^ mice both before and after infection (Supplementary Fig. 5a–d). We therefore performed additional analyses focused on monocytes. GO term analysis of monocytes revealed that monocytes from control mice exhibited greater expression of genes associated with cell chemotaxis and leukocyte apoptotic processes in response to *S. aureus* infection. In contrast, the genes that were less specifically associated with antibacterial response programs were enriched in the monocytes from Pdgfra/Ccl2fl/fl mice. A UMAP plot revealed 10 clusters (Supplementary Fig. 6a). Additionally, cluster 4 was reduced in Pdgfra/Ccl2^fl/fl^ mice (Supplementary Fig. 6b) and was associated with response-to-bacterium pathways, including Cd74-related programs (Supplementary Fig. 6c–e). These observations are consistent with those of the in vitro macrophage analyses and suggest that loss of fibroblast-derived CCL2 is associated with impaired antigen processing and antigen presentation programs in monocytes.

The results of the cell‒cell interaction analysis during *S. aureus* infection further revealed reduced interactions between fibroblasts and other cell types and decreased interactions among fibroblasts (Supplementary Fig. 7a), similar to observations in uninfected samples (Fig. 4E). We therefore performed additional analyses focused on fibroblasts. GO terms revealed that in control mice with *S. aureus* infection, enriched GO terms were associated with extracellular matrix organization. In contrast, Pdgfra/Ccl2^fl/fl^ mice exhibited enrichment of genes related to ATP metabolic processes (Supplementary Fig. 7b). Further analysis of the fibroblast clusters revealed an increased proportion of cluster 2 genes in Pdgfra/Ccl2^fl/fl^ mice (Supplementary Fig. 7c, d), which included genes involved in ATP metabolism during infection (Supplementary Fig. 7e, f).

**Fig. 5:**
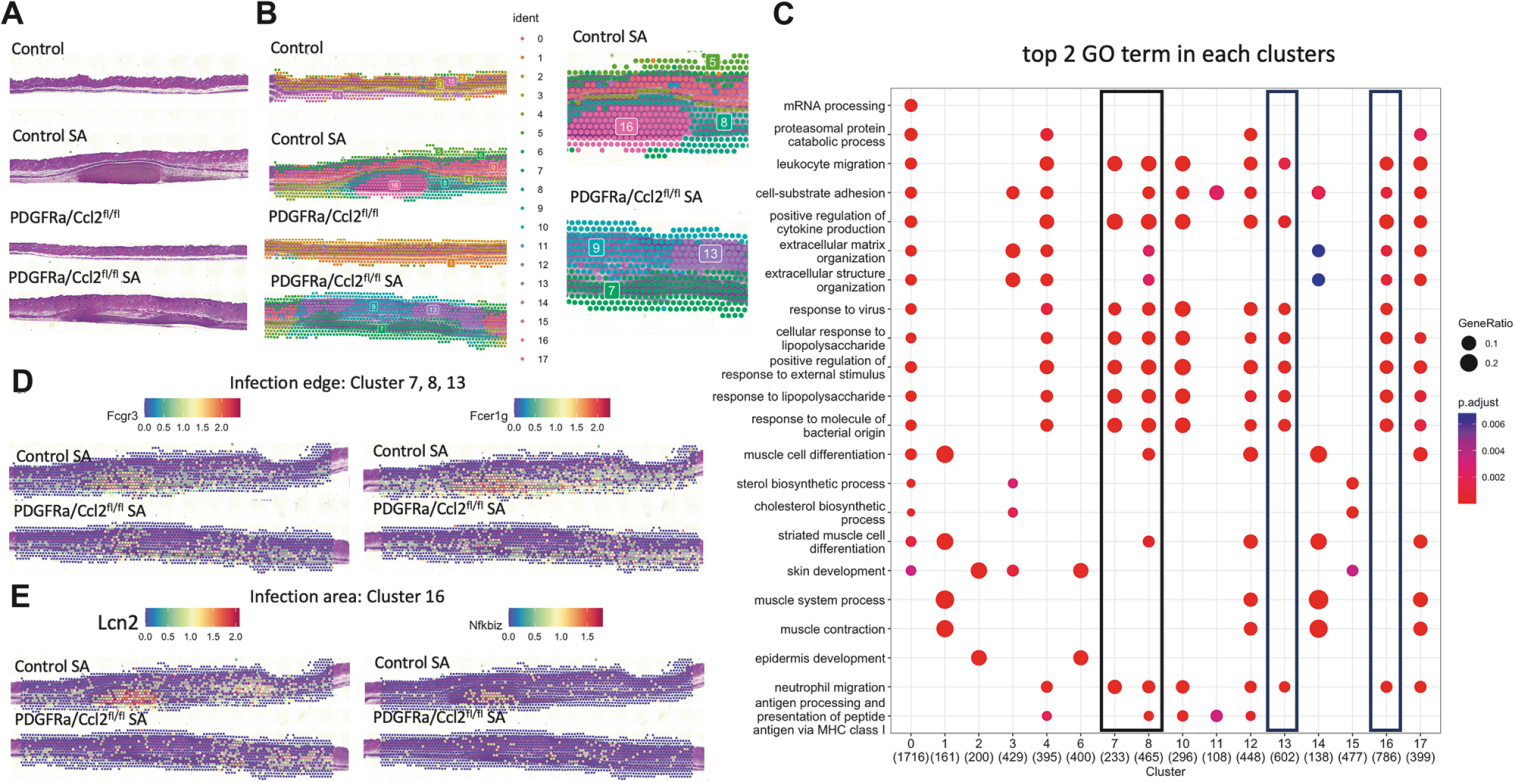
Fibroblast-derived CCL2 contributes to the spatial organization of the transcriptional response to *S. aureus* infection. Spatial transcriptomic analysis was performed on skin from the control, Pdgfra/Ccl2^fl/fl^, *S. aureus* infection, and Pdgfra/Ccl2^fl/fl^ + *S. aureus* infection groups on day 3 after infection. **A** Representative H&E-stained sections used for spatial transcriptomic analysis. **B** Spatial cluster maps showing transcriptionally distinct regions across experimental groups. **C** The top 2 GO terms associated with spatial clusters in each group. **D** Spatial feature plots of representative genes enriched at the infection edge, including Fcgr3 and Fcer1g. **E** Spatial feature plots of representative genes enriched within the infected lesion, including Lcn2 and Nfkbiz. Scale bar: 50 microns. Statistical significance was determined using Student’s unpaired two-sided t test (**B**). Error bars indicate the mean ± SEM; * *P* < 0.05, ** *P* < 0.01, *** *P* < 0.001. Each experiment was repeated at least three times

Since the local response to invasive *S. aureus* infection in the skin is highly spatially organized, we next performed spatial transcriptomic analysis of mouse skin three days post-infection. Across the control, *S. aureus*-infected (SA), Pdgfra/Ccl2^fl/fl^, and Pdgfra/Ccl2^fl/fl^ SA samples, 18 distinct transcriptomic clusters were identified (Fig. 5A, B and Supplementary Fig. 8a, b). In control SA mice, six concentric transcriptomic layers were arranged around the infection site (clusters 16 → 8, 4, 0, 6, and 5), suggesting a coordinated, multilayered response program. In contrast, in Pdgfra/Ccl2^fl/fl^ SA mice, this spatial organization was altered, with only three clusters (9, 7, and 13) surrounding the lesion (Fig. 5B). GO term analysis further revealed that the infection-edge clusters in Pdgfra/Ccl2^fl/fl^ (Cluster 7) were associated with reduced extracellular matrix organization and antigen presentation pathways, including reduced expression of *Fcgr3* and *Fcer1g* (Fig. 5C, D and Supplementary Fig. 8c; 9). Moreover, the expression of inflammatory mediators such as Lcn2 and Nfkbiz increased in the fibroblasts within the infected lesions of control SA mice (Cluster 16), whereas this response was reduced in Pdgfra/Ccl2^fl/fl^ mice (Fig. 5E). Together, these findings suggest that fibroblast-derived CCL2 contributes to the spatial organization of the infection-edge transcriptional response and supports the activity of stromal and immune-associated programs linked to extracellular matrix remodeling and antigen presentation.

### Fibroblast-derived Ccl2 regulates immune responses in macrophages and fibroblasts

**Fig. 6:**
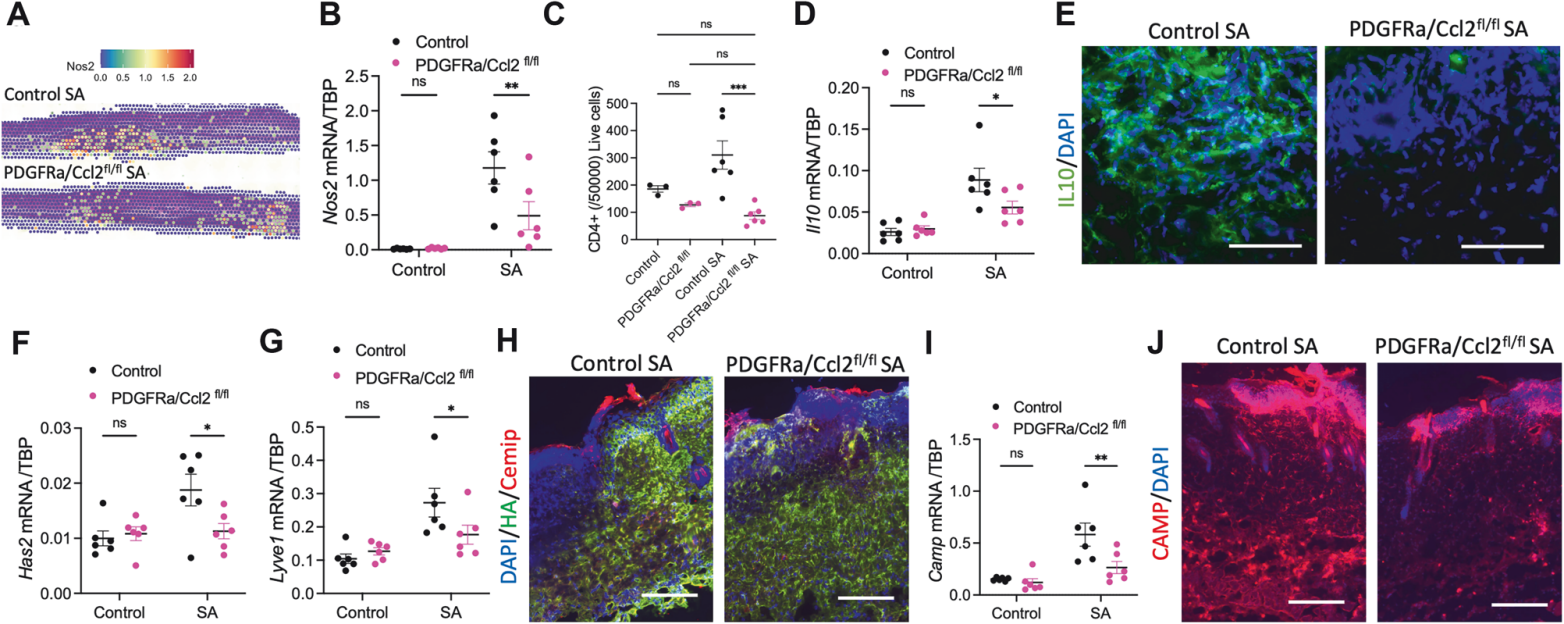
Fibroblast-derived CCL2 regulates macrophage and fibroblast immune responses. Control and Pdgfra/Ccl2^fl/fl^ mice were analyzed after intradermal *S. aureus* infection. **A** Spatial feature plot of Nos2 expression. **B** qPCR analysis of Nos2 expression in whole skin on day 3 after infection. **C** Number of CD4+ cells in infected skin on day 3. **D** qPCR analysis of Il10 expression on day 6 after infection. **E** Immunofluorescence staining of IL10 on day 6 after infection. **F, G** qPCR analysis of Has2 and Lyve1 expression on day 3 after infection. **H** Immunofluorescence staining of HABP and Cemip on day 3 after infection. **I** mRNA expression of Camp after 3 days of infection. **J** Immunofluorescence staining of CAMP on day 3 after infection. Scale bar: 50 microns. Statistical significance was determined using ordinary two-way ANOVA and Sidak’s multiple comparisons two-sided test (**D**). The error bars indicate the mean ± SEM; * *P* < 0.05, ** *P* < 0.01, *** *P* < 0.001. Each experiment was repeated at least three times

On the basis of our observations that the transcriptional response of Pdgfra/Ccl2^fl/fl^ during *S. aureus* infection included changes in communication with several cell types that participate in defense against infection, we next focused on analyzing these cells to better understand how they may contribute to the increased susceptibility to *S. aureus* infection. Nitric oxide (NO) production is known to play a critical role in host defense by macrophages [24, 25]. Compared with that in control mice, the number of Nos2 mRNA signals detected by spatial sequencing decreased in Pdgfra/Ccl2^fl/fl^ mice (Fig. 6A), and qPCR of whole skin from the site of infection also revealed fewer Nos2 in Pdgfra/Ccl2^fl/fl^ mice after infection (Fig. 6B). Flow cytometry analysis revealed a decreased number of CD4+ lymphocytes after *S. aureus* infection in Pdgfra/Ccl2^fl/fl^ mice (Fig. 6C). No difference in the number of CD45-positive cells, neutrophils, or macrophages was observed between control and Pdgfra/Ccl2^fl/fl^ mice with or without infection (Supplementary Fig. 10a–c). However, compared with control Pdgfra/Ccl2^fl/fl^ mice, Pdgfra/Ccl2fl/fl mice presented a small increase in CD11c+ and MHCII+ dendritic cells and an increase in IL10 staining after infection (Fig. 6D, E and Supplementary Fig. 10d, e).

scRNAseq and spatial sequencing analysis also revealed decreased expression of genes associated with ECM organization in Pdgfra/Ccl2^fl/fl^ mice. Decreases in the mRNA levels of hyaluronan synthase 2 (Has2) and lymphatic vessel endothelial hyaluronan receptor 1 (*Lyve1*) and total hyaluronan were observed in Pdgfra/Ccl2^fl/fl^ mice compared with controls after SA infection (Fig. 6F, G). No increase in the expression of cell migration-inducing and hyaluronan-binding protein (*Cemip*), the enzyme responsible for hyaluronan degradation during skin injury, was detected (Fig. 6H and Supplementary Fig. 10f).

As fibroblasts primarily express hyaluronan in the dermis [26, 27] and are activated to undergo adipogenesis to defend against SA infection through the production of the antimicrobial peptide cathelicidin (*Camp)* [2], we next evaluated *the* expression of Camp. Pdgfra/Ccl2^fl/fl^ mice expressed significantly less *Camp* in the skin after SA (Fig. 6I, J and Supplementary Fig. 10g). This decrease in *Camp* expression was not associated with a decrease in the number of neutrophils but was associated with decreased Camp staining in Pdgfra+ fibroblasts (Supplementary Fig. 10h, j).

### Ccl2 is inducible in dermal fibroblasts and is required for adipogenesis

**Fig. 7:**
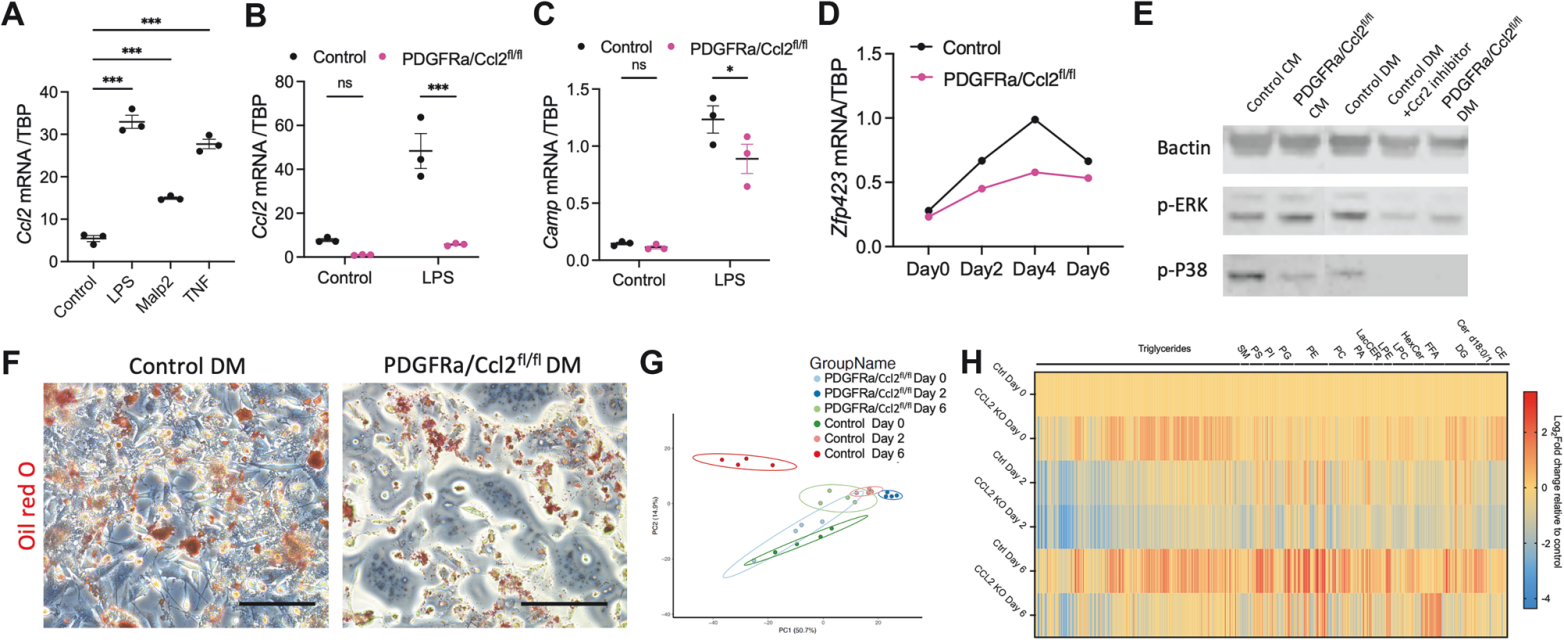
Ccl2 promotes adipocyte differentiation and lipid remodeling in dermal fibroblasts. Mouse primary fibroblasts (mFbs) were isolated from the back skin of control and Pdgfra/Ccl2^fl/fl^ mice and analyzed in vitro. **A** qPCR analysis of Ccl2 expression in control fibroblasts after stimulation with LPS, Malp2, or TNF. **B** qPCR confirmation of Ccl2 deficiency in fibroblasts from PDGRRa/Ccl2^fl/fl^ mice. **C** qPCR analysis of cAMP expression after LPS stimulation. **D** qPCR analysis of Zfp423 expression during adipocyte differentiation after treatment with differentiation medium (DM) at days 0, 2, 4, and 6. **E** Western blotting of phosphorylated ERK and p38 after DM treatment in the presence of a CCR2 inhibitor or in fibroblasts from Pdgfra/Ccl2^fl/fl^ mice. **F** Lipid accumulation during adipocyte differentiation was assessed by Oil red O staining. DMS-based shotgun lipidomic analysis of mFB culture supernatant. **G** DMS-based shotgun lipidomic analysis of fibroblasts after DM treatment. PCA plot of lipid composition. **H** Quantification of representative classes of lipids altered during adipocyte differentiation. CE cholesterol esters, Cer d18:1 ceramides, DG diacylglycerols, Cer d18:0 dihydroceramides, FFA free fatty acids, HexCER hexosyl ceramides, LacCER lactosyl ceramides, LPC lysophosphatidylcholine, LPE lysophosphatidylethanolamine, PA phosphatidic acid, PC phosphatidylcholine, PE phosphatidylethanolamine, PG phosphatidylglycerol, PI phosphatidylinositol, PS phosphatidylserine, SM sphingomyelin. Scale bar: 50 microns. Statistical significance was determined using ordinary one-way ANOVA and Tukey’s multiple comparison two-sided test and ordinary two-way ANOVA and Sidak’s multiple comparisons two-sided test (d). The error bars indicate the mean ± SEM; * *P* < 0.05, ** *P* < 0.01, *** *P* < 0.001. Each experiment was repeated at least 3 times

To better understand how Ccl2 is induced in fibroblasts during infection and why the expression of the antimicrobial peptide Camp is reduced in Pdgfra/Ccl2^fl/fl^ mice, we next evaluated mouse primary dermal fibroblasts in vitro. Ligands for TLR4 (LPS), TLR2/6 (Malp2), and TNF each induced Ccl2 expression and secretion in fibroblasts derived from wild-type mice (Fig. 7A and Supplementary Fig. 3d). Fibroblasts derived from Pdgfra/Ccl2^fl/fl^ mice were confirmed to lack Ccl2 expression (Fig. 7b). Consistent with the in vivo findings, fibroblasts derived from Pdgfra/Ccl2^fl/fl^ mice showed decreased *Camp* expression after LPS activation (Fig. 7C) and decreased expression of the adipocyte differentiation marker zinc finger protein 423 (Zfp423) following the addition of adipocyte differentiation medium (DM) (Fig. 7D). Further evidence that CCL2 influenced adipocyte differentiation was observed in the decreased phosphorylation of ERK and p38 after DM following the addition of a CCR2 inhibitor or in fibroblasts from Pdgfra/Ccl2^fl/fl^ mice treated with DM (Fig. 7E Supplementary Fig. 11a, b).

Compared with control fibroblasts, fibroblasts derived from Pdgfra/Ccl2^fl/fl^ mice also demonstrated decreased lipid accumulation during adipocyte differentiation (Fig. 7F and Supplementary Fig. 11c). In addition, inhibition of Ccl2, ERK, or p38 decreased Camp expression (Supplementary Fig. 12a–d). DMS-based shotgun lipidomic analysis further demonstrated that the lipids produced by Pdgfra/Ccl2^fl/fl^ fibroblasts differed from those produced by control cells when they were stimulated to undergo adipocyte differentiation (Fig. 7G). Notably, cholesterol ester-related species and lipid classes associated with neutral lipid storage were consistently reduced, together with altered ceramide profiles (Fig. 7H).

Together, these findings indicate that CCL2 promotes ERK/p38-dependent adipogenic differentiation and is associated with a broader lipid remodeling program linked to the antimicrobial and barrier-supportive functions of fibroblasts.

To extend these findings to human cells, we next examined human primary adipogenic dermal fibroblasts (HPADs). Adipocyte differentiation medium (DM) increased the expression of CAMP, ADIPOQ, and PPARG2, whereas pharmacologic inhibition of CCL2, CCR2, p38, or ERK attenuated these responses (Supplementary Fig. 13a–c). Consistent with these transcriptional changes, DM increased lipid accumulation, as determined by BODIPY staining, and this effect was reduced by CCL2 or CCR2 inhibition (Supplementary Fig. 13d, e). To test whether fibroblast-derived CCL2 also modulates human monocyte responses, CD14+ cells were treated with conditioned medium from HPADs transfected with scramble control or two independent siCCL2 constructs. Knockdown of CCL2 in HPADs was confirmed at the mRNA level (Supplementary Fig. 13f), and conditioned medium from siCCL2-treated HPADs reduced CD74 and IL1B expression in CD14+ cells (Supplementary Fig. 13g, h). Flow cytometric analysis also revealed a reduced proportion of CD74 + CD14+ cells under these conditions (Supplementary Fig. 13i).

## Discussion

In this study, we show that fibroblast-derived CCL2 contributes to host defense against *S. aureus* infection by modulating macrophage-associated immune responses and reactive adipogenesis. Recent research has increasingly highlighted the immune-regulatory roles of fibroblasts, challenging their traditional characterization as merely structural components of tissues. Building on this evolving perspective, we aimed to identify fibroblast-derived pathways that contribute to host defense and inflammation. By analyzing human and mouse single-cell sequencing datasets, we revealed that fibroblasts express a variety of cytokines and chemokines. In the steady state, CCL2, CXCL12, and IL-6 are among the inflammatory mediators prominently expressed by fibroblasts. Importantly, CCL2 expression was enriched in a specific subset of fibroblasts, whereas CXCL12 and IL-6 were expressed more broadly across the fibroblast population (Figs. 1 and 2). On the basis of these observations, we hypothesized that fibroblast-derived CCL2 plays a distinct role in immune regulation. In support of this hypothesis, our in vitro findings indicate that fibroblast-derived CCL2 modulates macrophage immune gene expression and antigen presentation-related programs (Fig. 3).

The importance of fibroblast-derived CCL2 was further supported by fibroblast-specific deletion of CCL2 in mice, which resulted in increased susceptibility to *S. aureus* infection (Figs. 4 and 5). This increase in susceptibility was accompanied by altered immune-associated responses in both monocytes and fibroblasts, supporting a role for fibroblast-derived CCL2 in coordinating innate immune defenses. Mechanistically, loss of fibroblast-derived CCL2 was associated with changes in monocyte-associated programs, reduced Nos2 expression, altered T-cell proliferation, and increased IL-10 staining during infection (Fig. 6). These findings suggest that fibroblast-derived CCL2 helps shape the immune environment during bacterial challenge, but they also report that this effect is multifactorial and not limited to a single downstream pathway.

Beyond these immune-associated effects, we also revealed a role for fibroblast-derived CCL2 in promoting fibroblast-to-adipocyte differentiation through a pathway associated with ERK and p38 signaling (Fig. 7 and Supplementary Figs. 11–13). In fibroblast differentiation assays, loss or inhibition of CCL2 signaling was associated with reduced expression of adipogenic and antimicrobial markers, including Camp, Adipoq, and Pparg2, as well as decreased lipid accumulation. In parallel, lipidomic analysis revealed altered lipid remodeling, including reductions in cholesterol ester-related species and broader changes in ceramide-associated profiles, which are consistent with impaired adipocyte maturation and altered inflammatory or antimicrobial functions. Together, these findings support a model in which fibroblast-derived CCL2 influences both adipogenic differentiation and lipid metabolic remodeling in fibroblasts. In addition, conditioned supernatants from fibroblasts with reduced CCL2 altered downstream responses in CD14+ cells, including reducing Il1b and CD74 expression, together with changing CD74-related surface phenotypes. These observations support a broader role for fibroblast-derived CCL2 in coordinating both stromal and monocyte/macrophage-associated immune programs.

Importantly, our findings do not suggest that CAMP is the sole effector downstream of fibroblast-derived CCL2. Rather, our data support that fibroblast-derived CCL2 coordinates host defense components, such as macrophage-associated immune programs and reactive adipogenesis with CAMP expression.

These findings broaden the current understanding of fibroblasts during bacterial infection. Our results suggest that fibroblasts integrate chemokine signaling, lipid remodeling, and antimicrobial programs within infected tissue. This may help explain how changes in the state of fibroblasts alter the local immune environment and susceptibility to infection.

A key limitation of this study is that although our data support roles for fibroblast-derived CCL2 in both macrophage-associated immune programs and stromal adipogenic–antimicrobial responses, we did not determine the relative contribution of these pathways to host defense against *S. aureus* infection in vivo. In addition, while our inhibitor and knockdown experiments support the involvement of the CCL2–CCR2–ERK/p38 axis, they do not fully establish pathway order or exclude additional downstream mediators. Future studies using rescue or cell-type-specific reconstitution approaches will be needed to define the dominant effector mechanisms and their hierarchy during infection.

In summary, our findings identify fibroblast-derived CCL2 as an important regulator of host defense against *S. aureus* infection, macrophage-associated immune programs, stromal adipogenic–antimicrobial responses, and leukocyte recruitment, thereby expanding the current understanding of fibroblast immune function

## Materials and methods

### Animals and animal care

All animal experiments were approved by the University of California, San Diego, Institutional Animal Care and Use Committee (Protocol No. S09074). For all the animal studies, the animals were randomly selected without formal prerandomization, and quantitative measurements were performed without the opportunity for bias.

C57BL/6 mice were purchased from The Jackson Laboratory. Pdgfra/Ccl2^fl/fl^ mice on the C57BL/6 background were crossbred and maintained at UCSD. The mice were housed under specific pathogen–free conditions with a 12-h light and 12-h dark cycle at 20–22°C and 30–70% humidity with unrestricted access to water and standard chow. Experimental and littermate control animals were age- and sex-matched 8–12-wk-old males and females, respectively.

### Bacterial strains

*S. aureus* strain USA300 is a predominant community-associated methicillin-resistant *S. aureus* (MRSA) strain, and AH4807, a USA300 MRSA strain containing the phage11::LL29luxCDABEG reporter plasmid, was tested in a manner that was similar to that previously described [28, 29] and was kindly provided by Alexander Horswill (Department of Immunology & Microbiology at the University of Colorado).

### Mouse model of *S. aureus* skin infection

Skin infection experiments were performed as described previously [30]. The ***S. aureus*** strain USA300/MRSA was used for infection. In brief, the backs of sex-matched and age-matched (8 weeks to 12 weeks) adult wild-type or Pdgfra/Ccl2^fl/fl^ mice were shaved, and the hair was removed by chemical depilation (Nair), after which the mice were injected intradermally with 100 μl of a medium-logarithmic growth phase of *S. aureus* (2 × 10^6^ CFU of bacteria) in PBS. The mice were sacrificed after 3- and 8-mm skin punch biopsies were performed, at which time the center of the injection site was harvested. Infected skin surrounding the infection center (6–8 mm) void of the center abscess was carefully dissected for RNA extraction. Skin biopsies were homogenized in 1 ml of TRIzol with 2 mm zirconia beads in a mini-bead beater 16 (Biospect, Bartlesville, OK). For in vivo live bacterial imaging, mice were imaged under isoflurane inhalation anesthesia (2%). Photons emitted from luminescent bacteria were collected during a 1 min exposure using the Xenogen IVIS Imaging System and Living Image software (Xenogen, Alameda, CA). Bioluminescent image data are presented on a pseudocolor scale (blue represents the least intense signal, and red represents the most intense signal) overlaid onto a grayscale photographic image. Using the image analysis tools in living image software, circular analysis windows (of uniform area) were overlaid onto regions of interest, and the corresponding bioluminescence values (total flux) were measured.

### Cell culture

For primary fibroblast studies, neonatal (P1) cells were used unless otherwise noted. Primary dermal fibroblasts were isolated by our laboratory as previously described (Zhang LJ et al., *Science* 2015;347:67–71) and used at passage 1. Cells were grown in Dulbecco’s modified Eagle’s medium (DMEM) supplemented with 10% FBS, GlutaMAX (35050061; Thermo Fisher Scientific), and antibiotic–antimycotic (15240062; Thermo Fisher Scientific). After 2 days, the confluent cells were stimulated with recombinant cytokines, purified toll ligands, or an adipogenesis-inducing cocktail. Adipogenesis was induced as described previously (Zhang LJ et al., *Science* 2015;347:67–71). The fibroblast culture supernatant was collected and added to fresh culture medium to achieve a final concentration of 20% for the chemotaxis assay and treatment of MHS macrophages. MHS macrophages were cultured under standard conditions in complete medium. For conditioned medium experiments, MHS macrophages were treated with control medium, control fibroblast-conditioned medium, or *Ccl2*-deficient fibroblast-conditioned medium. Fibroblast-conditioned medium was added to fresh culture medium at a final concentration of 20%. Cells were harvested after the indicated treatment period for RNA extraction and downstream RT‒qPCR or bulk RNA‒seq analysis.

Human preadipocytes (HPADs) were cultured and induced to differentiate. At the initiation of differentiation, cells were treated with the indicated inhibitors or neutralizing reagents targeting CCL2 (10 nM), CCR2 (1 nM), p38 (10 µM), or ERK (10 µM). Cells were collected on day 4 after induction of differentiation for RT‒qPCR analysis or fixed for lipid staining.

### Chemicals and reagents

Anti-CAMP antibodies were obtained from our laboratory as described previously [31]; BODIPY^®^ FL dye was purchased from Thermo Fisher (Houston, TX). HA-binding protein was purchased from Millipore. Lipopolysaccharide (LPS) solution (500X) was purchased from eBioscience™, Malp2 was purchased from Enzo Biochem Inc., and recombinant TNF was purchased from Fisher Scientific. Anti-CCL2 antibodies were purchased from Invitrogen (# 13-7096-85). Anti-CCR2 antibodies were purchased from Selleck (PF-4136309 (INCB8761, PF-04136309)). Adezmapimod (SB203580, RWJ 64809, PB 203580)) (a p38 inhibitor) was purchased from Selleck (S1076). FR 180204 (an ERK1/2 inhibitor) was purchased from Selleck (S7524). Silencer RNA for CCL2 was purchased from Invitrogen^TM^ (Silencer™ Select Validated siRNA, s12566 and s12567).

### Histology and immunohistochemistry (IHC)

Tissue biopsies were directly embedded in OCT compound or paraffin. Paraffin-embedded tissues were used for hematoxylin and eosin (H&E) staining, and frozen sections were fixed in 4% PFA for 20 min for immunofluorescence staining. For IHC, fixed and permeabilized frozen tissue sections were blocked with Image-iT FX reagent (Invitrogen) before they were incubated with anti-CAMP. Anti-IL10 and anti-Gr1 antibodies were purchased from Abcam (Cambridge, MA), and anti-Cemip antibody was provided by KAO. The samples were subsequently incubated with appropriate 488- or 568-conjugated secondary antibodies. The nuclei were counterstained with DAPI. All the images were taken with an Olympus BX41 microscope (widefield).

### Oil Red O staining

An Oil Red O stock solution was prepared at 3 mg/ml in 100% isopropanol. Following stimulation, the cells were washed three times with phosphate-buffered saline (PBS) and fixed in 10% formalin for 2 h at room temperature. The cells were then rinsed with 60% isopropanol. Oil Red O working solution (60% diluted in dH_2_O) was then added to the cells for 2 h and 30 min. Then, the cells were again rinsed three times with PBS.

### BODIPY staining and quantification

To analyze lipid accumulation during adipocyte differentiation, cells were fixed after the indicated differentiation period and stained with BODIPY FL dye. The nuclei were counterstained with DAPI. Fluorescence images were acquired under identical exposure settings across the experimental groups. BODIPY fluorescence intensity was quantified using Fiji/ImageJ under identical thresholding and measurement settings across groups. The signal intensity was normalized to the analyzed area or cell number, as indicated in the figure legend.

### Western blotting

Mouse colon tissues were homogenized in RIPA buffer (Thermo Fisher). After centrifugation, the cell lysates were subjected to SDS‒PAGE and transferred onto polyvinylidene difluoride membranes (IPVH 00010; Millipore). In brief, the membranes were blocked in blocking buffer (LICOR Biosciences), after which they were incubated with antibodies against p-ERK, p-p38 and β-Actin overnight at 4 °C. The membranes were analyzed by immunoblotting with the indicated antibodies.

### Tissue processing for single-cell RNA sequencing

Tissue samples from 3 mice in each group were minced with a razor blade into 1 cm fragments, suspended in enzymatic digestion buffer collagenase and DNase I as previously described, incubated with frequent agitation at 37 °C for 120 min, and triturated briefly with a 5 ml pipet. Cells in a single-cell suspension were then passed through a 100-micron mesh filter. Then, dead cells were removed using a Dead Cell Removal kit (Miltenyi Biotec, 130-090-101) following the manufacturer’s instructions. Live cells were manually counted using a hemocytometer and resuspended in 0.04% ultrapure BSA (Thermo Fisher, AM2618). A total of 20,000 live cells were loaded on the 10X Genomics Chromium system.

### Tissue processing for spatial transcriptomics

Skin tissues from untreated and *S. aureus*-infected control and Pdgfra/Ccl2^fl/fl^ mice were fixed in cold 4% PFA, embedded in paraffin, sectioned at 4 µm, and placed on glass slides. The experimental slide with the tissue was fixed and stained with hematoxylin and eosin (H&E) and imaged using a Keyence BZX-700 fluorescence microscope (Keyence) at 4X magnification and transferred to a sequencing slide by Visium CytAssist. Sequence libraries were then processed according to the manufacturer’s instructions (10x Genomics, Visium Spatial Transcriptomic).

### Library construction protocol

Single-cell suspensions were loaded onto the 10X Genomics Chromium Controller instrument to generate single-cell GEMs. GEM-RT and library construction were performed following the 10X Genomics Protocol. Library fragment size distributions were determined using an Agilent Bioanalyzer High Sensitivity Chip, and library DNA concentrations were determined using a Qubit 2.0 fluorometer (Invitrogen). Libraries were sequenced using an Illumina NovaSeq platform.

### Data analysis

For mouse skin samples, the 10X Genomics Cell Ranger version 7.2 software pipeline with default parameters was used to perform sample demultiplexing, barcode processing, alignment to the mm10 reference genome, and single-cell gene counting. The data were further filtered, processed and analyzed using the Seurat R toolkit version 5. Integration anchors between datasets were identified using the FindIntegrationAnchors function (dims = 50) and integrated using the IntegrateData function (dims = 50). The integrated data were then scaled, and principal component analysis (PCA) was performed on highly variable features. Significant principal components (PCs) were identified using a combination of statistical and heuristic methods and were employed to guide clustering. Neighbors and clusters were identified using the FindNeighbors and FindClusters functions, respectively, and visualized using uniform manifold approximation and projection (UMAP) or t-distributed stochastic neighbor embedding (t-SNE). Cluster biomarkers were identified using the FindAllMarkers function (Wilcoxon rank sum test). Scored cells were projected onto UMAP, and cells were color-coded on the basis of their score. Cells present in each cell cycle phase were also quantified. CellChat analysis. The R package CellChat [32] was used to quantitatively infer and analyze intercellular communication networks on the basis of our single-cell RNA sequencing data. CellChat employs network analysis and pattern recognition methodologies to predict major signaling inputs and outputs for cells, as well as how these cells and signals coordinate for various functions. One of the key functions of CellChat is its ability to classify signaling pathways and delineate conserved and context-specific pathways through manifold learning and quantitative contrast.

### Data analysis by BioTuring

The human single-cell sequencing dataset (GSE201333) was obtained, processed, and analyzed by the online platform BioTuring. A total of 500 K cells were analyzed, and 67,540 Pdgfra-high cells were extracted as fibroblasts. These cells are reclustered, visualized using UMAP, and pseudotime analyzed.

### Flow cytometry analyses

Skin samples collected from control and Pdgfra/Ccl2^fl/fl^ mice with or without *S. aureus* infection were cut into small pieces, digested with 2.5 mg/mL collagenase D and 30 ng/mL DNAse1 for 2 h at 37°C, and then filtered through a 70 µm filter to generate single-cell suspensions for FACS analyses. The cells were then stained with Fixable Viability Dye eFluor™ 506 (eBioscience, 65-0866-14) and blocked with anti-mouse CD16/32 (eBioscience, 14016185), followed by staining with antibody cocktails for immune cells. The antibody cocktail for immune cells included Brilliant Violet 711™-CD45 (BioLegend, 103147), PECy7-CD11b (BioLegend, 101216), FITC-Ly6G (eBioscience, 11593182), PE-F4/80 (eBioscience, 12480182), APC-CD11C (BioLegend, 117310), AF700-MHCII (eBioscience, 56532182), and APC-Cy7-CD3 (BioLegend, 100222). For flow cytometric analysis of human cells, PBMCs were harvested by Lymphoprep (Cosmobio, 1114544). Cells were stained with fluorophore-conjugated antibodies against CD14 (BD Biosciences, 555397) and CD74 (BioLegend, 326807). Cells were first gated on live single cells, after which CD14-positive cells were identified. CD74 expression was then assessed within the CD14-positive population, and the percentage of CD74-positive cells among CD14-positive cells was quantified. FACS analyses for surface expression of immune cell markers were performed by the BD FACSCanto RUO machine and analyzed by FlowJo V10 software.

### Reverse transcription‒quantitative PCR (RT‒qPCR) analyses

RT‒qPCR was used to determine mRNA abundance. Total cellular RNA was extracted using the PureLink RNA Mini Kit (Life Technologies Corporation). One hundred nanograms of mRNA was reverse transcribed to cDNA using a Verso cDNA Synthesis Kit (Thermo Fisher Scientific, Inc.). Quantitative real-time PCR was performed on a CFX96 real-time system (Bio-Rad) using a predeveloped Taqman gene expression assay (Applied Biosystems) or SYBR Green mix (Bimake, Houston, TX). The housekeeping gene Tbp (TATA-binding box protein) was used to normalize gene expression in the samples. The specific primer sequences are shown in Table 1.

**Table 1:** PCR primer sequences

Gene name		Sequence
Mouse		
Tbp	forward	CCTTGTACCCTTCACCAATGAC
Tbp	reverse	ACAGCCAAGATTCACGGTAGA
ll1b	forward	GAAATGCCACCTTTTGACAGTG
ll1b	reverse	TGGATGCTCTCATCAGGACAG
Nos2	forward	GTTCTCAGCCCAACAATACAAGA
Nos2	reverse	GTGGACGGGTCGATGTCAC
Cd74	forward	AGTGCGACGAGAACGGTAAC
Cd74	reverse	CGTTGGGGAACACACACCA
Il10	forward	CGGGAAGACAATAACTGCACCC
Il10	reverse	CGGTTAGCAGTATGTTGTCCAGC
Has2	forward	GGTCCAAGTGCCTTACTGAAAC
Has2	reverse	TGTACAGCCACTCTCGGAAGTA
Lyve1	forward	ACCAGGTAGAGTCAGCGCAGAA
Lyve1	reverse	CAGGACACCTTTGCCATTCTTCC
Camp	forward	CAAGGAACAGGGGGTGG
Camp	reverse	TCCGGCTGAGGTACAAGT TT
Ccl2	forward	AGGTCCCTGTCATGCTTCTG
Ccl2	reverse	TCTGGACCCATTCCTTCTTG
Zfp423	forward	CAAGAGGAGAGAAATGAGGACGA
Zfp423	reverse	AGTGATCGCAGGTGTAAATTGAC
Human		
TBP	forward	TGTATCCACAGTGAATCTTGGTTG
TBP	reverse	GGTTCGTGGCTCTCTTATCCTC
CCL2	forward	CAGCCAGATGCAATCAATGCC
CCL2	reverse	TGGAATCCTGAACCCACTTCT
CAMP	forward	AGGTCCTCAGCTACAAGGAAG
CAMP	reverse	TCTTGAAGTCACAATCCTCTGGT
PPARG	forward	GCCCAGGTTTGCTGAATGTG
PPARG	reverse	TGAGGACTCAGGGTGGTTCAG
ADIPOQ	forward	GCTCAGCATTCAGTGTGGGA
ADIPOQ	reverse	GTACAGCCCAGGAATGTTGC
CD74	forward	ACCGAAAGTACTGACCAA
CD74	reverse	TGGAGTGGCAGATAGTTG
IL1B	forward	AGCTACGAATCTCCGACCAC
IL1B	reverse	CGTTATCCCATGTGTCGAAGAA

### Lipidomic analysis

Lipidomic analyses were conducted at the UCLA Lipidomics Core following their established protocol, as previously outlined. Briefly, for tissue samples, 50–100 mg of frozen dermis layers of skin were placed in a 2 mL homogenizer tube preloaded with 2.8 mm ceramic beads (Omni #19–628). PBS was added to the tube, and the sample was homogenized in an Omni Bead Ruptor Elite (3 cycles of 10 s at 5 m/s with a 10-s dwell time). For lipid extraction, 3–6 mg of tissue homogenate was transferred to a glass tube for lipid extraction using a modified Bligh and Dyer extraction method [33]. Prior to biphasic extraction, an internal standard mixture comprising 70 lipid standards across 17 subclasses was added to each sample (AB Sciex 5040156, Avanti 330827, Avanti 330830, Avanti 330828, and Avanti 791642). Following two consecutive extractions, pooled organic layers were evaporated in a Thermo SpeedVac SPD300DDA using a ramp setting of 4 at 35°C for 45 min with a total run time of 90 min. Lipid samples were reconstituted in a 1:1 mixture of methanol and dichloromethane with 10 mM ammonium acetate and transferred to robust samples (Thermo Fisher Scientific, 10800107) for analysis. Samples were analyzed on a Sciex 5500 with a DMS device (Lipidyzer platform) utilizing an expanded targeted acquisition list encompassing 1450 lipid species across 17 subclasses. The differential mobility device on the Lipidyzer was calibrated with EquiSPLASH LIPIDOMIX (Avanti 330731). Data analysis was conducted on an in-house data analysis platform similar to the Lipidyzer Workflow Manager. The instrument method, encompassing the settings, tuning protocol, and multiple reaction monitoring (MRM) list, has been previously described [34]. Quantitative values were normalized to milligrams of tissue weight.

### Statistical analysis

Statistical analyses were performed with GraphPad Prism. All the statistical tests performed are indicated in the figure legends.

### Study approval

All animal experiments were approved by the University of California, San Diego, Institutional Animal Care (Protocol No. S09074).

## Supplementary information

Supporting information

supplemental figures

## Data Availability

The sequencing data generated and analyzed in this study have been deposited in the Gene Expression Omnibus under the accession numbers GSE227836, GSE330279, and GSE270712. Source data and unprocessed images are provided with this paper and its Supplementary Information, where applicable. All other data supporting the findings of this study are available from the corresponding author upon reasonable request.
