# Supplementary material for: Fibroblast-derived CCL2 orchestrates immune responses and defends against Staphylococcus aureus skin infection
Source: Cell Mol Immunol. 2026 Jun 22;23(8):972–82. doi: 10.1038/s41423-026-01442-7 (PMC13424308; doi:10.1038/s41423-026-01442-7)
Supplement: Supplementary file 1 — Supporting information [file 41423_2026_1442_MOESM1_ESM.docx]

**Supporting Information**

Fibroblast-derived CCL2 orchestrates immune responses and defends against Staphylococcus aureus skin infection

**Authors:** Tatsuya Dokoshi^1,2^†, Marta Palomo-Irigoyen^1,3^, Kazuki Dai^2^, Terumi Hashimoto^2^, Hiroaki Konishi^2^, Lauriane Hivert^1^, Michelle Bagood^1^, Hung Chan^1^, Samia Almoughrabie^1^, Yoshiyuki Nakamura^1,4^, Kellen J. Cavagnero^1^, Teruaki Nakatsuji^1^, Mikihiro Fujiya^2^, Richard L. Gallo^1^

**Affiliations:**

^1^ Department of Dermatology, University of California, San Diego, La Jolla, CA 92037, United States.

^2^ Department of Internal Medicine Division of Gastroenterology, Asahikawa Medical University, Asahikawa, Hokkaido, Japan

^3^Genes and Disease Laboratory, Department of Dermatology, Medical University of Vienna, Vienna, Austria

^4^Department of Dermatology, University of Tsukuba: Tsukuba, Ibaraki, JAPAN

* Dr. Tatsuya Dokoshi,

Department of Internal Medicine Division of Gastroenterology

Asahikawa Medical University, Asahikawa,

2-1-1-1 midorigaoka higashi, Asahikawa, Hokkaido, 078-8510

Telephone: 0166-68-2462

Fax: 0167-45-4578

**Email:**  [ta1983@asahikawa-med.ac.jp](mailto:ta1983@asahikawa-med.ac.jp)

**This file includes:**

Figure legends of S1 to S8

Original image from western blotting

**Fig. S1. Chemokine expression analysis in a multi-organ scRNA-seq dataset.**

**(a**) Percentage of cells expressing candidate cytokines and chemokines across multiple cell types in a human scRNA-seq dataset. (**b**) Marker genes of mouse fibroblast clusters.

**Fig. S2. Mouse skin and colon fibroblasts contain distinct Cxcl12-, Il6-, and Ccl2-expressing subsets.**

Mouse fibroblasts were extracted from published single-cell RNA-seq datasets and reanalyzed.
(a) Relative proportion of fibroblast clusters identified from mouse skin and colon datasets.
(b) UMAP visualization of extracted fibroblasts.
(c–e) Feature plots showing expression of Cxcl12 (c), Il6 (d), and Ccl2 (e) in skin and colon fibroblasts.

**Fig. S3. Analyses of fibroblast-conditioned medium and macrophage responses..**

**(**a) Total number of recruited cells in the chemotaxis assay. (**b, c**) Relative abundance of Neutrophils(**b)** and Lymphocytes(**c**) among recruited cells**.** (**d**) CCL2 concentration in fibroblast-conditioned medium measured by ELISA. (**e**) Principal component analysis (PCA) of bulk RNA-seq data from MHS macrophages treated with the indicated media. (**f**) qPCR analysis of Cd74 expression in MHS macrophages treated with a CCL2 inhibitor. Statistical significance was determined using ordinary one-way ANOVA and Tukey’s multiple comparison two-sided test. Error bars indicate mean ± SEM; * P<0.05, ** P < 0.01, *** P<0.001. Each experiment was repeated at least 3 times

**Fig. S4. In vivo response to *S. aureus* infection in control and Pdgfra/Ccl2^fl/fl^ mice.**

(**a**) IVIS imaging of *S. aureus* on mouse back skin 3 days after injection. (**b**) luminescence intensity of *S. aureus* showing bacterial abundan in Pdgfra/Ccl2^fl/fl^ mice. (**c**) Relative abundance of cell types based on scRNASeq from Control and Pdgfra/Ccl2^fl/fl^. (**d**) UMAP plot of single-cell RNA-seq data from control, Pdgfra/Ccl2^fl/fl^, *S. aureus* infection, and Pdgfra/Ccl2^fl/fl^ + *S. aureus* infection groups. (**e**) Heatmap of representative differentially expressed genes across the indicated groups. Statistical significance was determined using ordinary one-way ANOVA and Tukey’s multiple comparison two-sided test. Error bars indicate mean ± SEM; * P<0.05, ** P < 0.01, *** P<0.001. Analysis by IVIS was repeated 3 times.

**Fig. S5. Cell–cell communication analysis in control and Pdgfra/Ccl2^fl/fl^ mice**

(**a**) Ranking of significant signaling pathways based on differences in overall information flow between inferred communication networks in control and Pdgfra/Ccl2^fl/fl^. Pathways enriched in control are shown in red, those equally represented in both groups in black, and those enriched in Pdgfra/Ccl2^fl/fl^. (**b**) Ranking of significant signaling pathways based on differences in overall information flow between control mice with S. aureus infection (control SA) and Pdgfra/Ccl2^fl/fl^ mice with infection (Pdgfra/Ccl2^fl/fl^_SA). Pathways enriched in control SA are shown in red, those equally represented in both groups in black, and those enriched in Pdgfra/Ccl2^fl/fl^_SA in green. (**c**) Heatmap summarizing signaling pathways contributing to outgoing and incoming communication in control and Pdgfra/Ccl2^fl/fl^. The color scale indicates relative signaling strength across cell types. Bar plots represent the total signaling strength for each cell type or pathway. (**d**) Heatmap summarizing signaling pathways contributing to outgoing and incoming communication in control and Pdgfra/Ccl2^fl/fl^ mice following by SA.

**Fig. S6. Transcriptomic analysis of monocyte populations.**

(**a**) UMAP plot of extracted monocyte cluster. (**b**) Relative abundance of Control, PDGFRa/Ccl2^fl/fl^, S. Aureus infection (control SA), and PDGFRa/Ccl2^fl/fl^ SA in Monocyte Cluster. (**c**) Top 2 GO terms in each monocyte cluster. (**d**) Top 3 differentially expressing genes in each monocyte cluster. (**e**) Violin plot and UMAP plot of Cd74 expression.

**Fig. S7. scRNASeq analysis of control and Pdgfra/Ccl2^fl/fl^ mice during *S. aureus* skin infection.**

(**a**) Heatmap showing the putative ligand–receptor interactions between cell types in control SA and Pdgfra/Ccl2^fl/fl^ mice after SA infection. The color bar represents the relative signaling strength of a signaling pathway across cell types. The bars indicate the sum of the signaling strength of each cell type or pathway. (**b**) Top 3 GO terms in each group. (**c**) UMAP plot of pooled groups with extracted fibroblast clusters. (**d**) UMAP plot of extracted fibroblast clusters in each experimental group. (**e**) Top 3 GO terms in each fibroblast cluster. (**f**) Top 3 differentially expressing genes in each cluster.

**Fig. S8. Spatial transcriptomic analysis of control and Pdgfra/Ccl2^fl/fl^ mice during *S. aureus* skin infection.**

(a) The spatial representation of clusters in control, Pdgfra/Ccl2^fl/fl^, *S. aureus* infection (control SA), and Pdgfra/Ccl2^fl/fl^ SA. (**b**) UMAP plot of spatial sequencing from control, Pdgfra/Ccl2^fl/fl^*, S. aureus* infection (SA), and Pdgfra/Ccl2^fl/fl^ SA. (**c**) Top 3 marker genes for each cluster.

**Fig. S9. Spatial mapping of transcriptomic clusters.**

Spatial plot showing the localization of identified clusters from the UMAP plot onto the corresponding spatial transcriptomic sequencing slide.

**Fig. S10. Flow cytometry and immunostaining analysis of mouse back skin following *S. aureus* infection.**

(**a**) The number of CD45+ cells in 50000 live cells. (**b**) CD11b+ and Ly6G+ Neutrophils. (**c**) CD11b+ and F4/80+ Macrophages. (**d**) CD11c+ and MHCII+ Dendritic Cells. (**e**) Quantification of IL-10 fluorescence intensity in control SA and Pdgfra/Ccl2^fl/fl^ SA. (related to Fig. 6e). (**f**) Quantification of HABP fluorescence intensity in control SA and Pdgfra/Ccl2^fl/fl^ SA. (related to Fig. 6h) (**g**) Quantification of Camp fluorescence intensity in control SA and Pdgfra/Ccl2^fl/fl^ SA. (related to Fig. 6j). (**h**) Immunofluorescence staining of Gr1 after 3 days of infection. (**i**) Immunofluorescence staining of Camp/Gr1/DAPI after 3 days of infection at high magnification of dermis. (**j**) Immunofluorescence staining of Camp/PDGFRa/DAPI after 3 days of infection at high magnification of dermis. (Scale bar: 25 microns. Camp: Red, Gr1: Green, PDGFRa: Green, DAPI: Blue) Statistical significance was determined using ordinary one-way ANOVA and Tukey’s multiple comparison two-sided test. Error bars indicate mean ± SEM * P<0.05, ** P < 0.01, *** P<0.001. Each experiment was repeated at least 3 times.

**Fig. S11. CCL2 promotes** adipocyte **differentiation and lipid remodeling in dermal fibroblasts.**

(**a, b**) Quantification of phosphorylated ERK(a) and p38(b) after DM treatment in the presence of a CCR2 inhibitor or in fibroblasts from Pdgfra/Ccl2^fl/fl^ mice. (related to Fig. 7e). (**c**) Quantification of Oil Red O staining intensity in control SA and Pdgfra/Ccl2^fl/fl^ SA. (related to Fig. 7f). Statistical significance was determined using ordinary one-way ANOVA and Tukey’s multiple comparison two-sided test. Error bars indicate mean ± SEM * P<0.05, ** P < 0.01, *** P<0.001. Each experiment was repeated at least 3 times.

**Fig. S12. CCL2 promotes camp production in mouse dermal fibroblasts.**

(**a**) Immunofluorescence staining of CAMP at day 2 after initiation of differentiation in control cells and cells treated with a Ccl2 inhibitor (iCcl2). (**b**) Quantification of camp after DM treatment in the presence of the indicated inhibitors. (**c**) Immunofluorescence staining of CAMP at day 2 after initiation of differentiation in control cells and cells treated with a p38 inhibitor (ip38) or ERK inhibitor (iErk). (**d**) Quantification of camp after DM treatment in the presence of the indicated inhibitors.Scale bar: 50 microns. Statistical significance was determined using ordinary one-way ANOVA and Tukey’s multiple comparison two-sided test. Error bars indicate mean ± SEM * P<0.05, ** P < 0.01, *** P<0.001. Each experiment was repeated at least 3 times.

**Fig. S13.** **CCL2 promotes adipogenic differentiation and regulates monocyte responses in human preadipocytes.**

Human preadipocytes (HPADs) were analyzed in vitro. (**a**) qPCR analysis of *CAMP* expression at day 4 after initiation of differentiation in control cells and cells treated with a CCL2 inhibitor (iCCL2), a CCR2 inhibitor (iCCR2), a p38 inhibitor (ip38), or an ERK inhibitor (iERK). (**b, c**) qPCR analysis of *ADIPOQ* (**b**) and *PPARG2* (**c**) expression at day 4 after initiation of differentiation. (**d**) Lipid accumulation during adipocyte differentiation assessed by BODIPY staining. BODIPY, green; DAPI, blue. (**e**) Quantification of BODIPY fluorescence intensity.

HPADs were transfected with two independent siRNAs targeting *CCL2* (siCCL2-1 and siCCL2-2). (**f**) qPCR analysis of *CCL2* expression 24 h after siRNA transfection. CD14 positive cells were isolated from human peripheral blood mononuclear cells (PBMCs) using magnetic beads and treated with conditioned medium from HPADs transfected with control siRNA or si*CCL2*. (**g, h**) qPCR analysis of *CD74* (**g**) and *IL1B* (**h**) expression in CD14 positive cells. (**i**) Flow cytometric quantification of the percentage of CD74 positive cells among CD14 positive cells after treatment with conditioned medium from HPADs transfected with control siRNA or si*CCL2*. Scale bar: 50 microns. Statistical significance was determined using ordinary one-way ANOVA and Tukey’s multiple comparison two-sided test. Error bars indicate mean ± SEM * P<0.05, ** P < 0.01, *** P<0.001. Each experiment was repeated at least 3 times.

**b-actin**

**
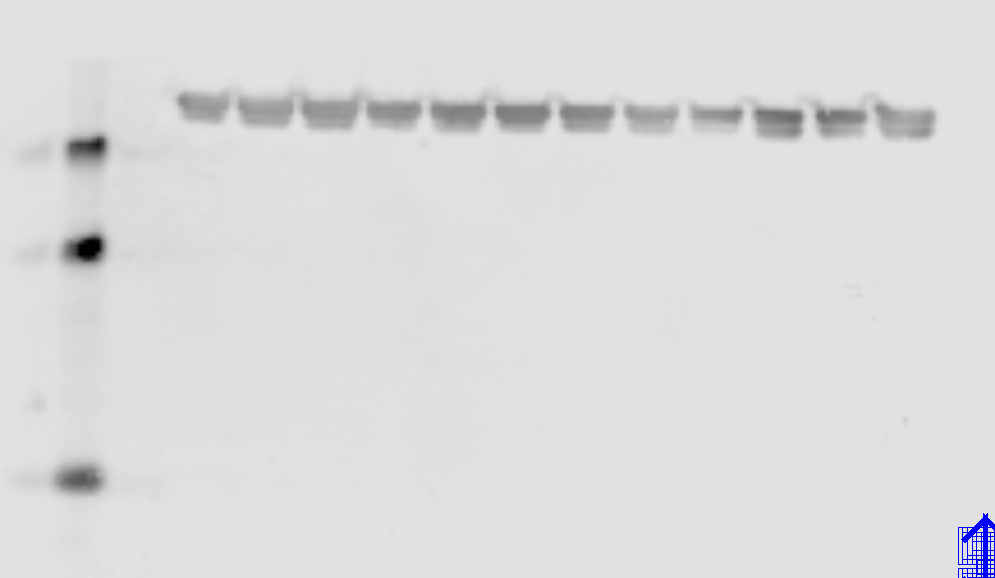
**

**p-p38**

**
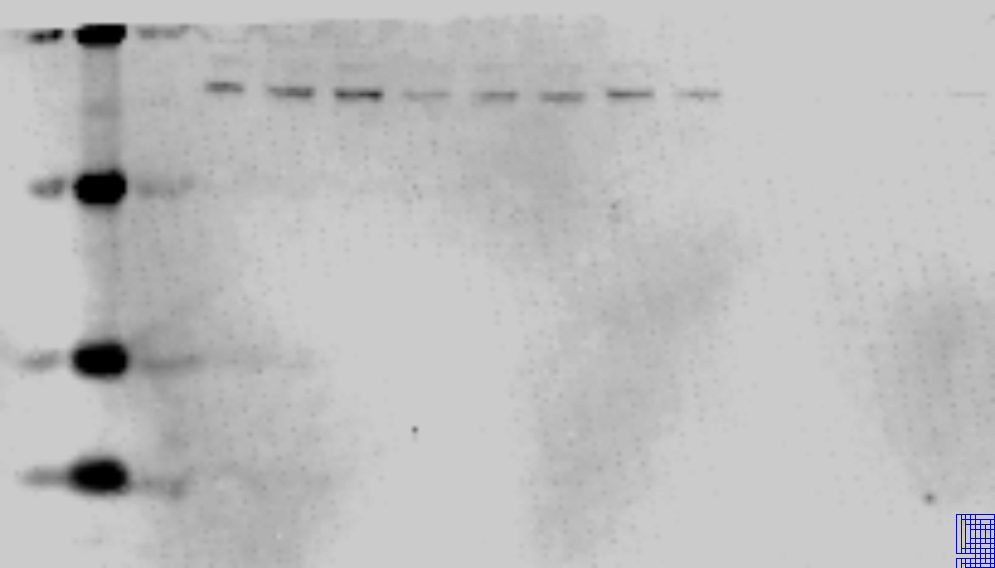
**

**p-Erk**

**
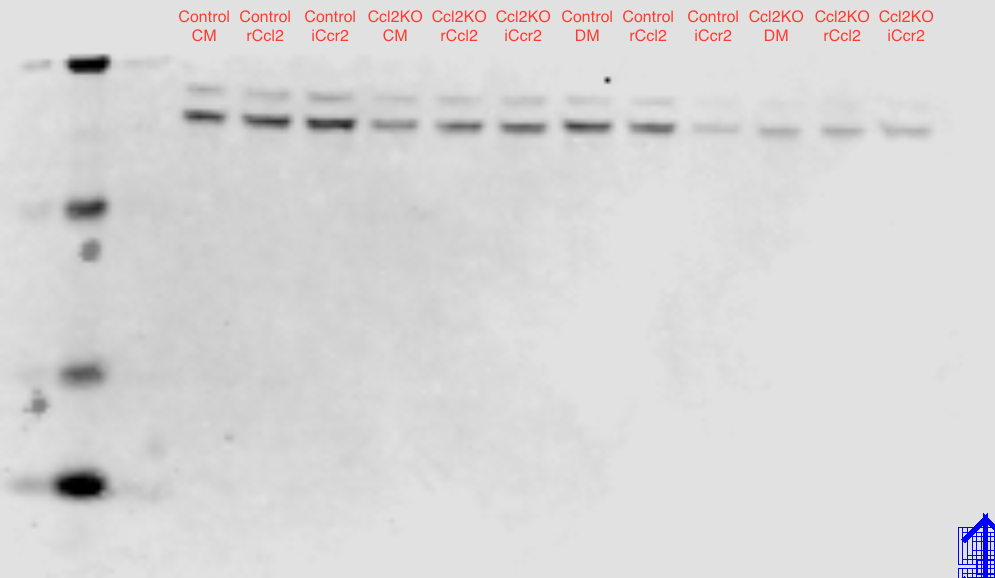
**
