# Supplementary material for: Fibroblast-derived CCL2 orchestrates immune responses and defends against Staphylococcus aureus skin infection
Source: Cell Mol Immunol. 2026 Jun 22;23(8):972–82. doi: 10.1038/s41423-026-01442-7 (PMC13424308; doi:10.1038/s41423-026-01442-7)

Supplemental Figure 1

a

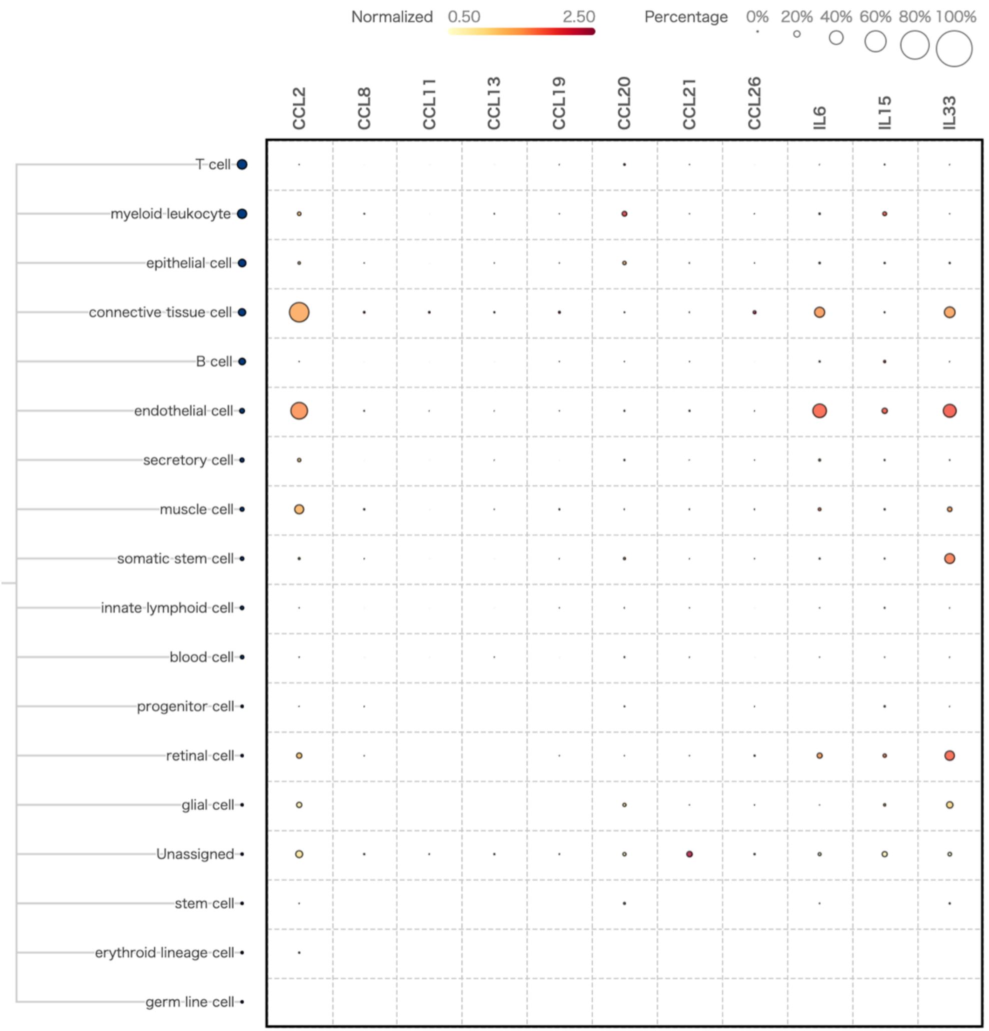

b

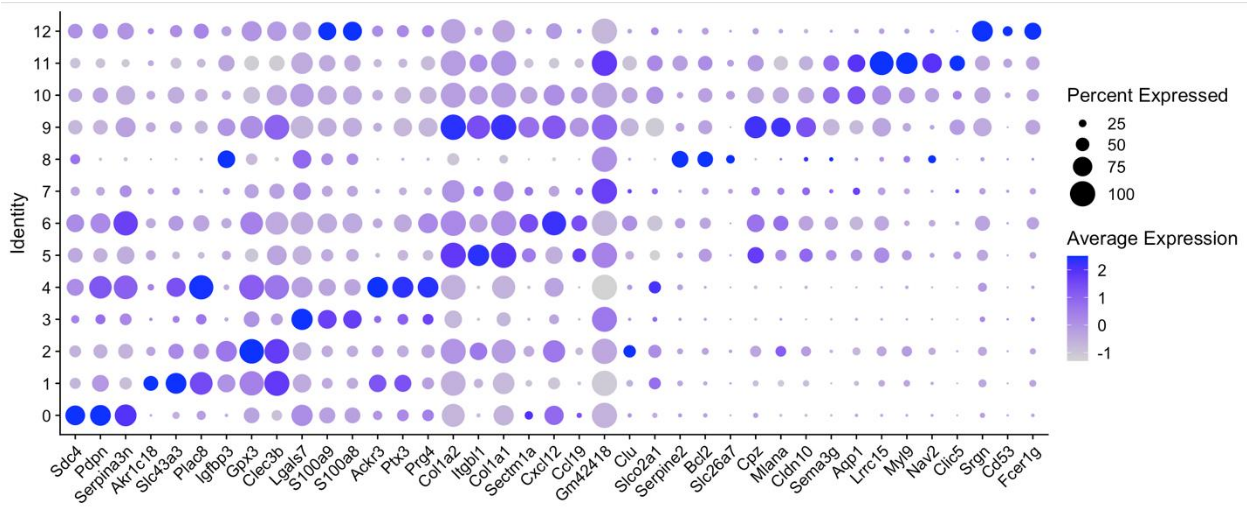

Supplemental Figure 2

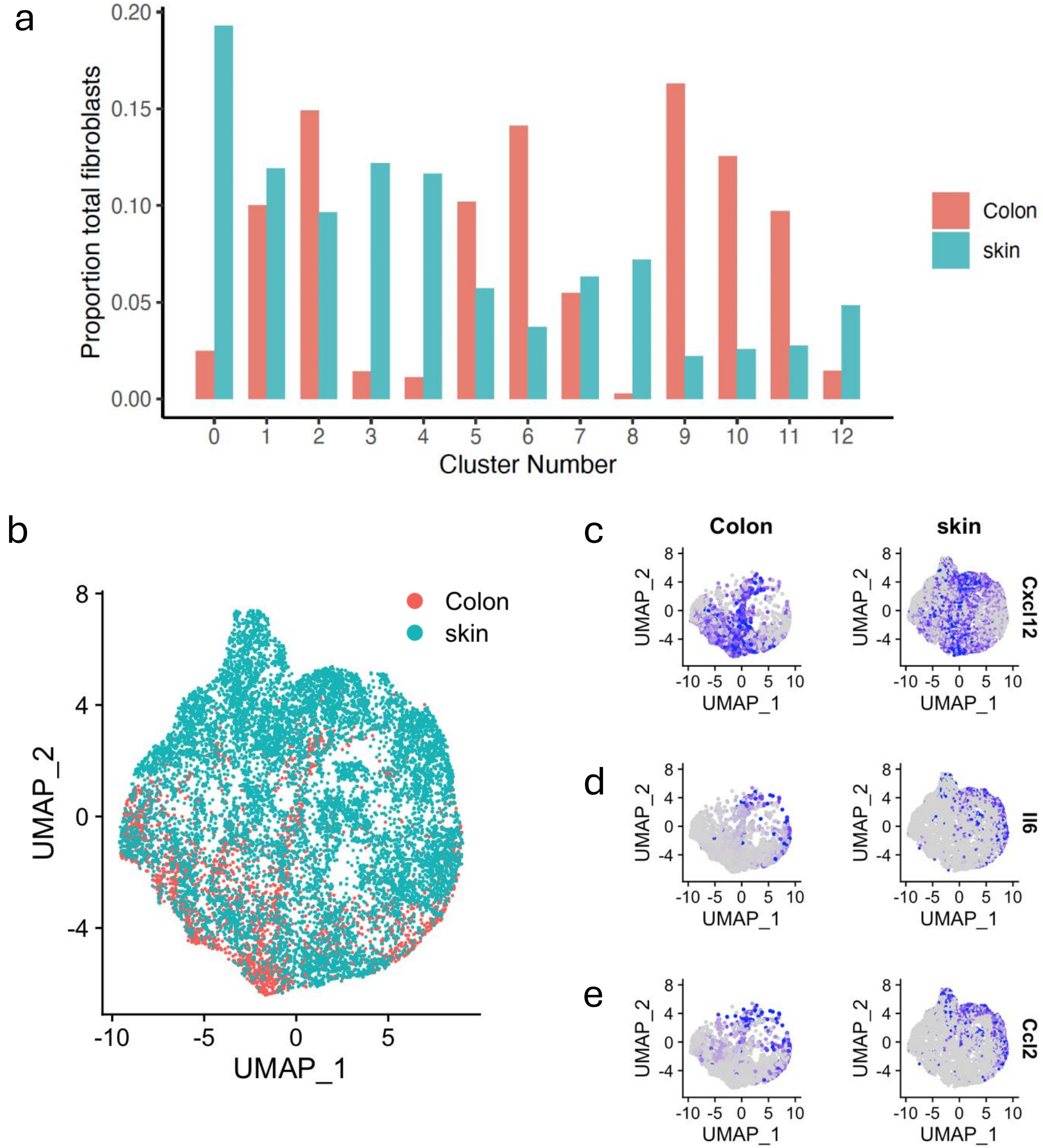

Supplemental Figure 3

migration assay

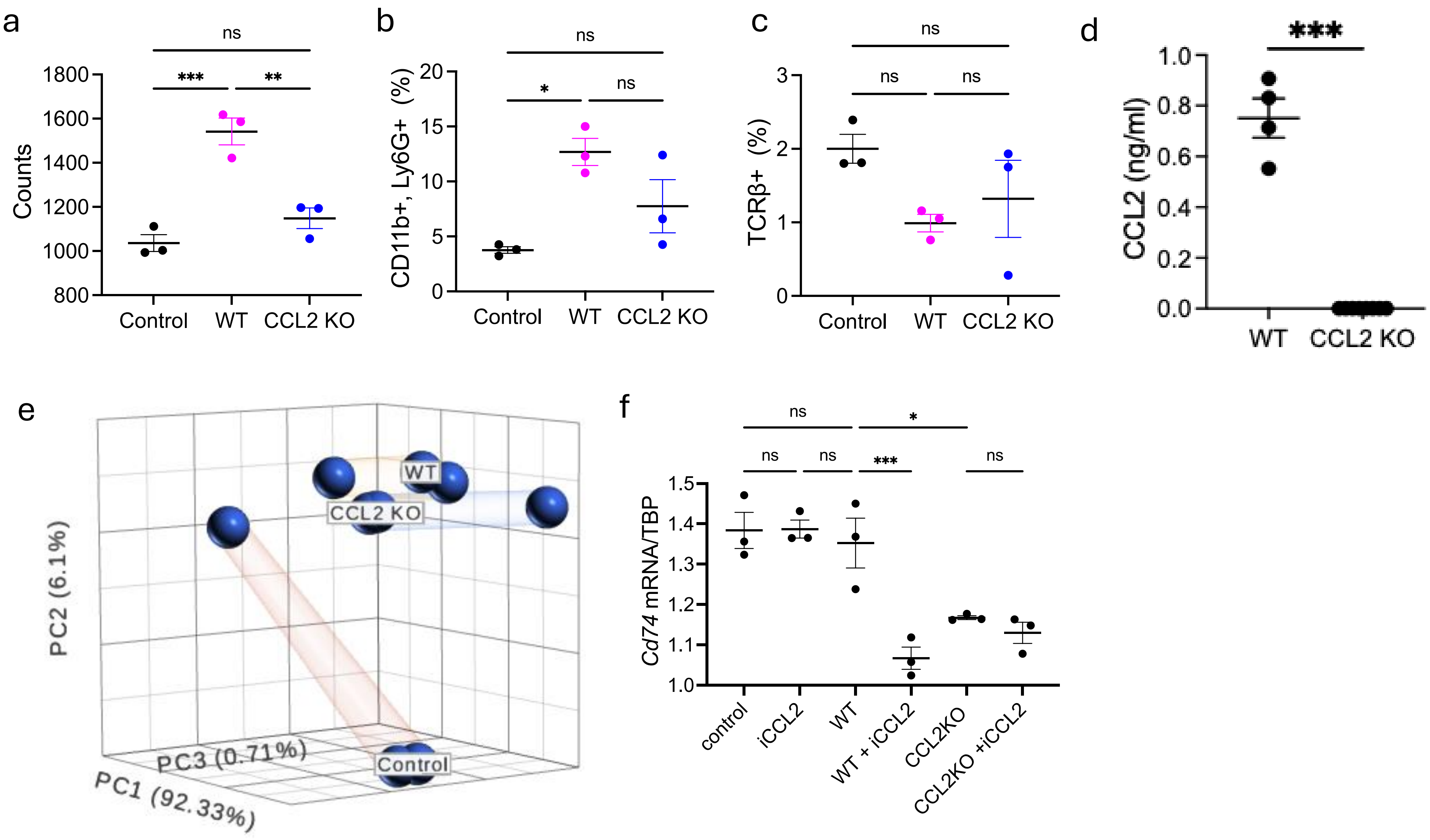

Supplemental Figure 4

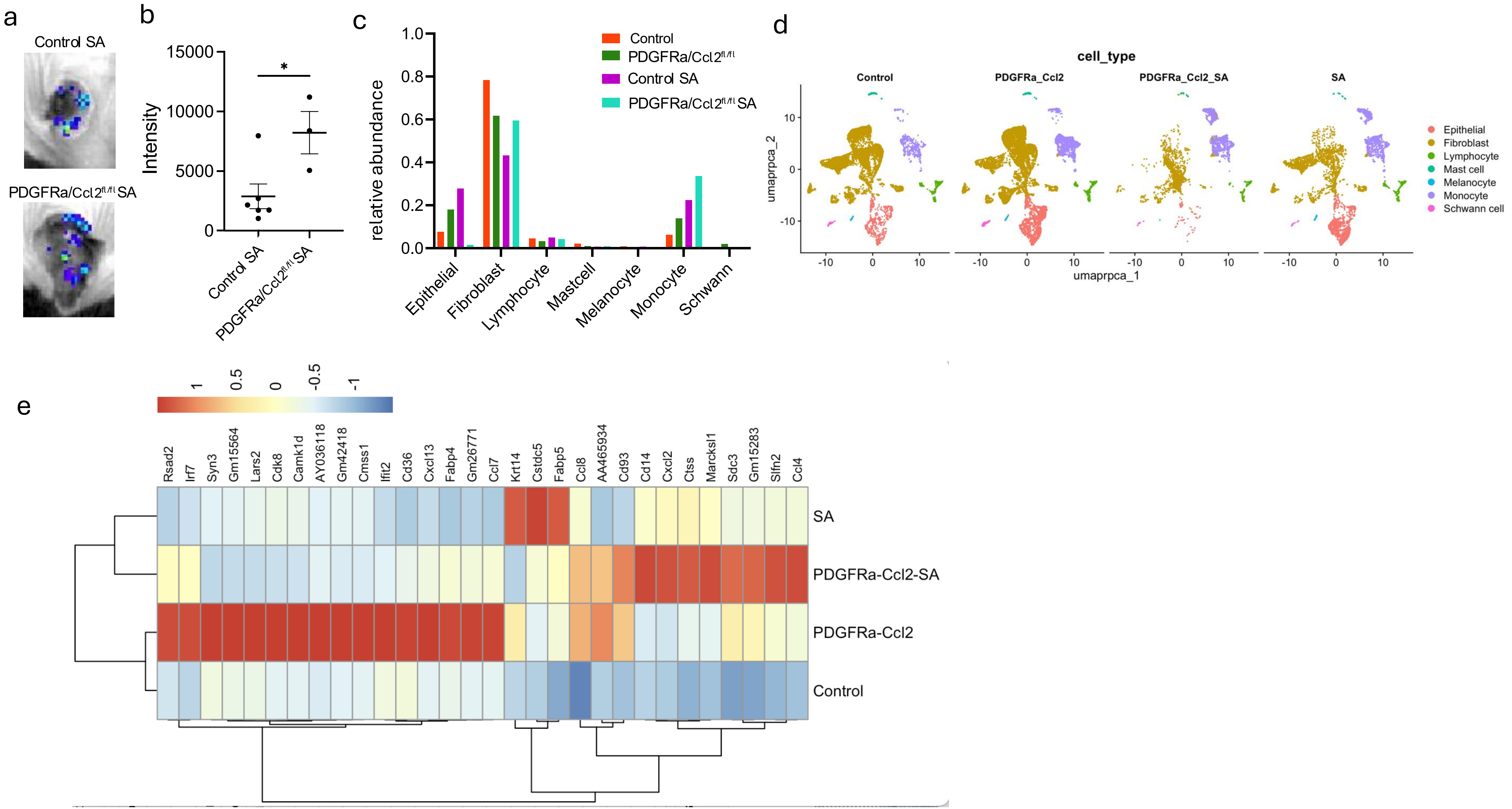

Supplemental Figure 5

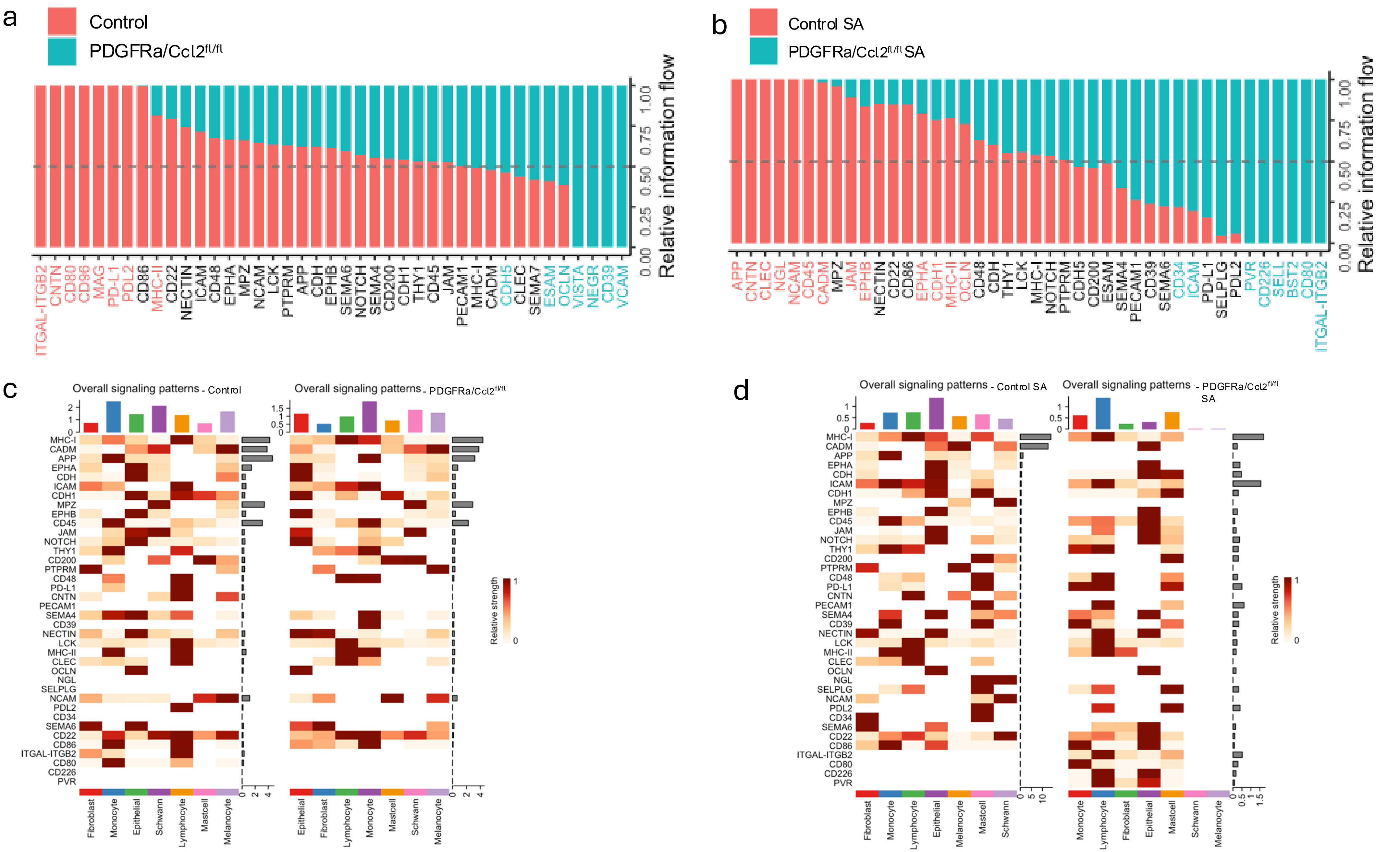

Supplemental Figure 6

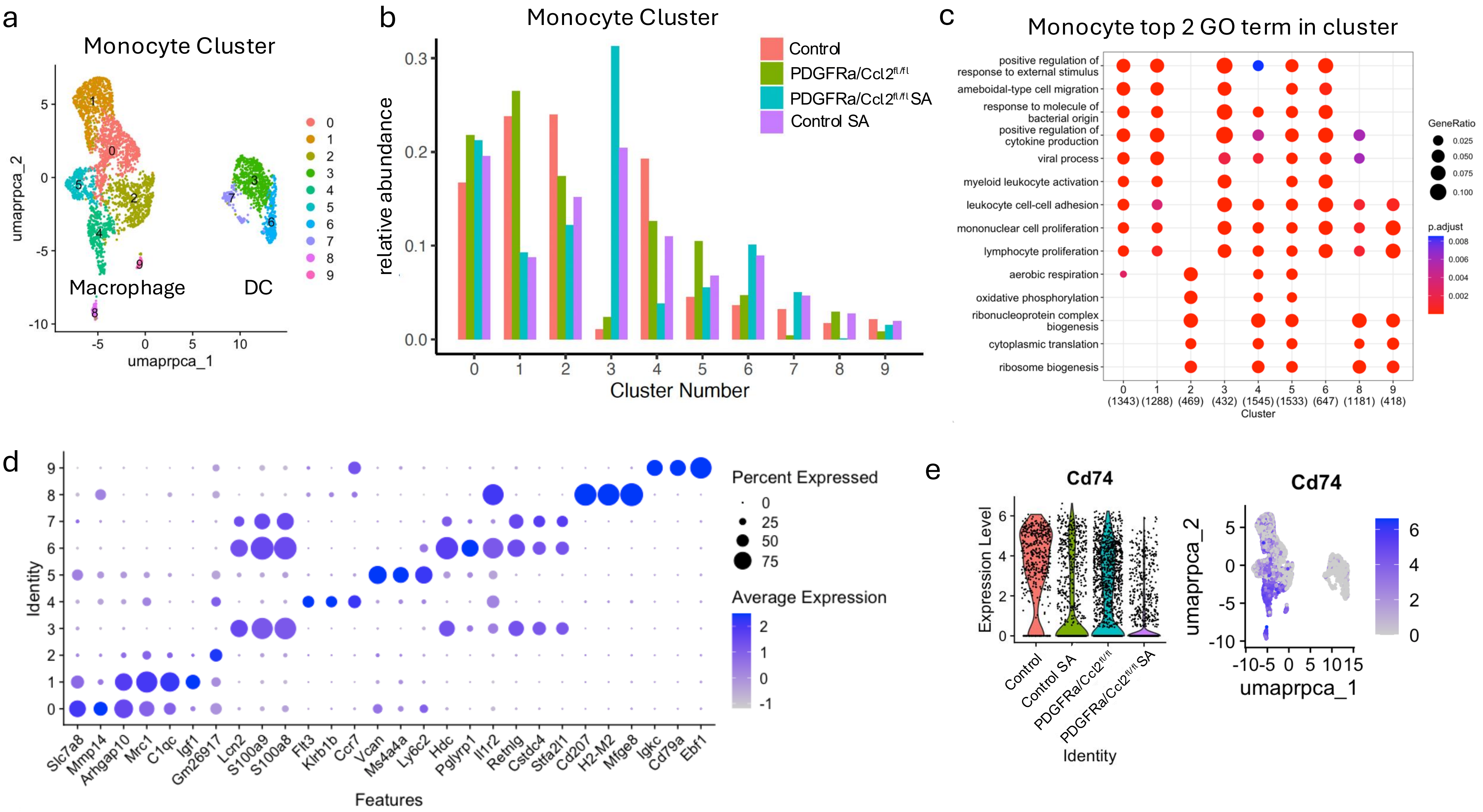

Supplemental Figure 7

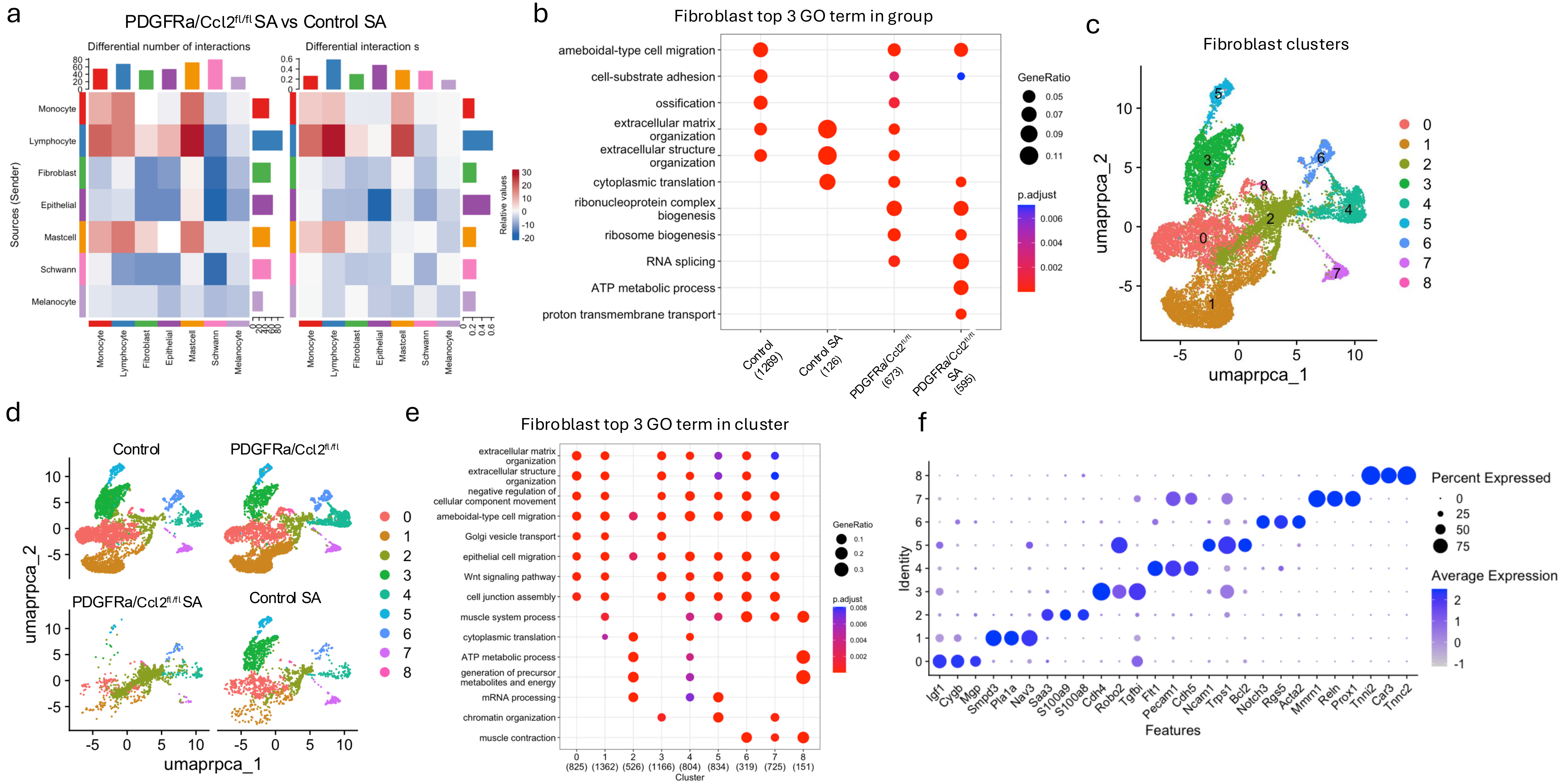

Supplemental Figure 8

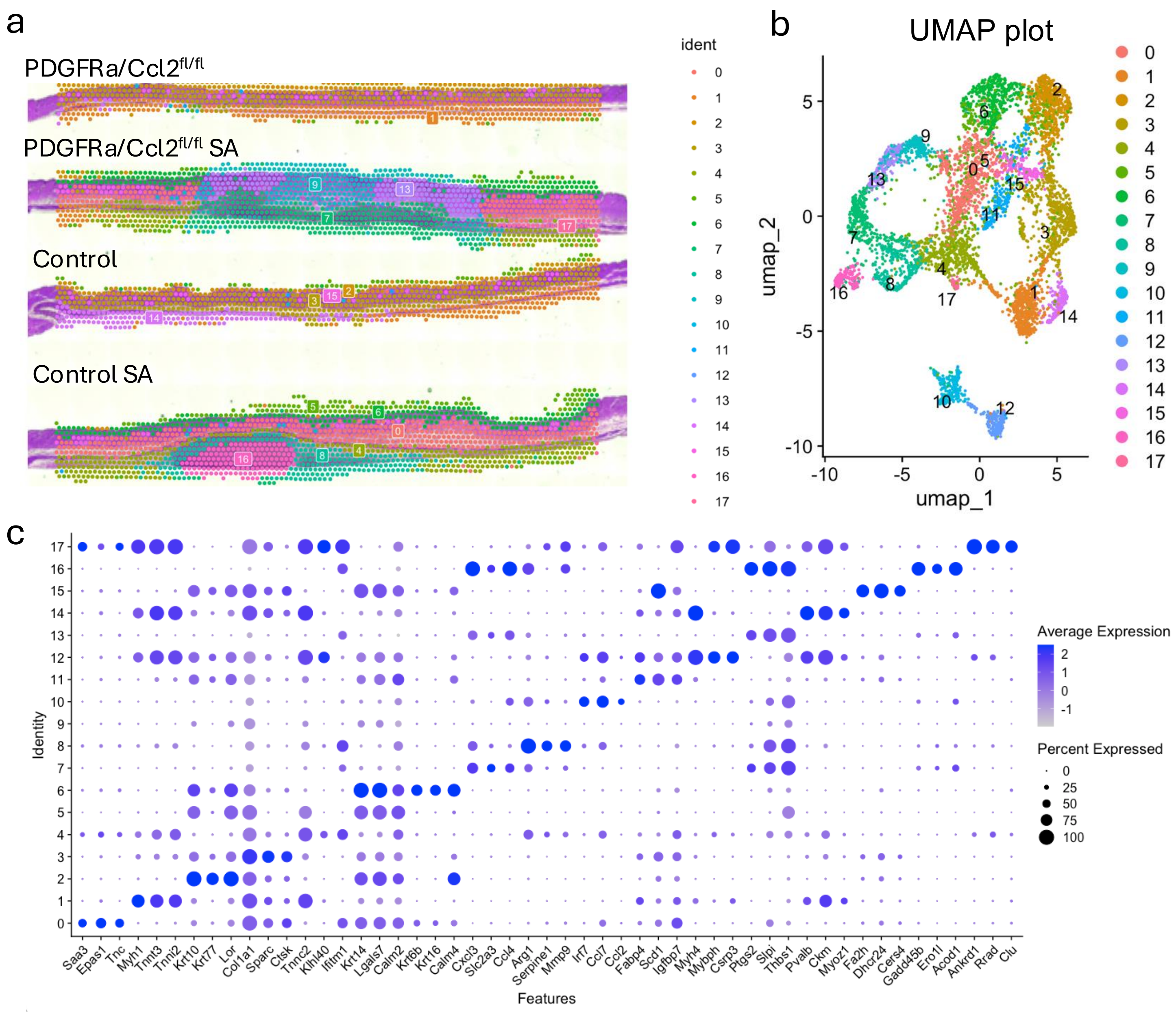

Supplemental Figure 9

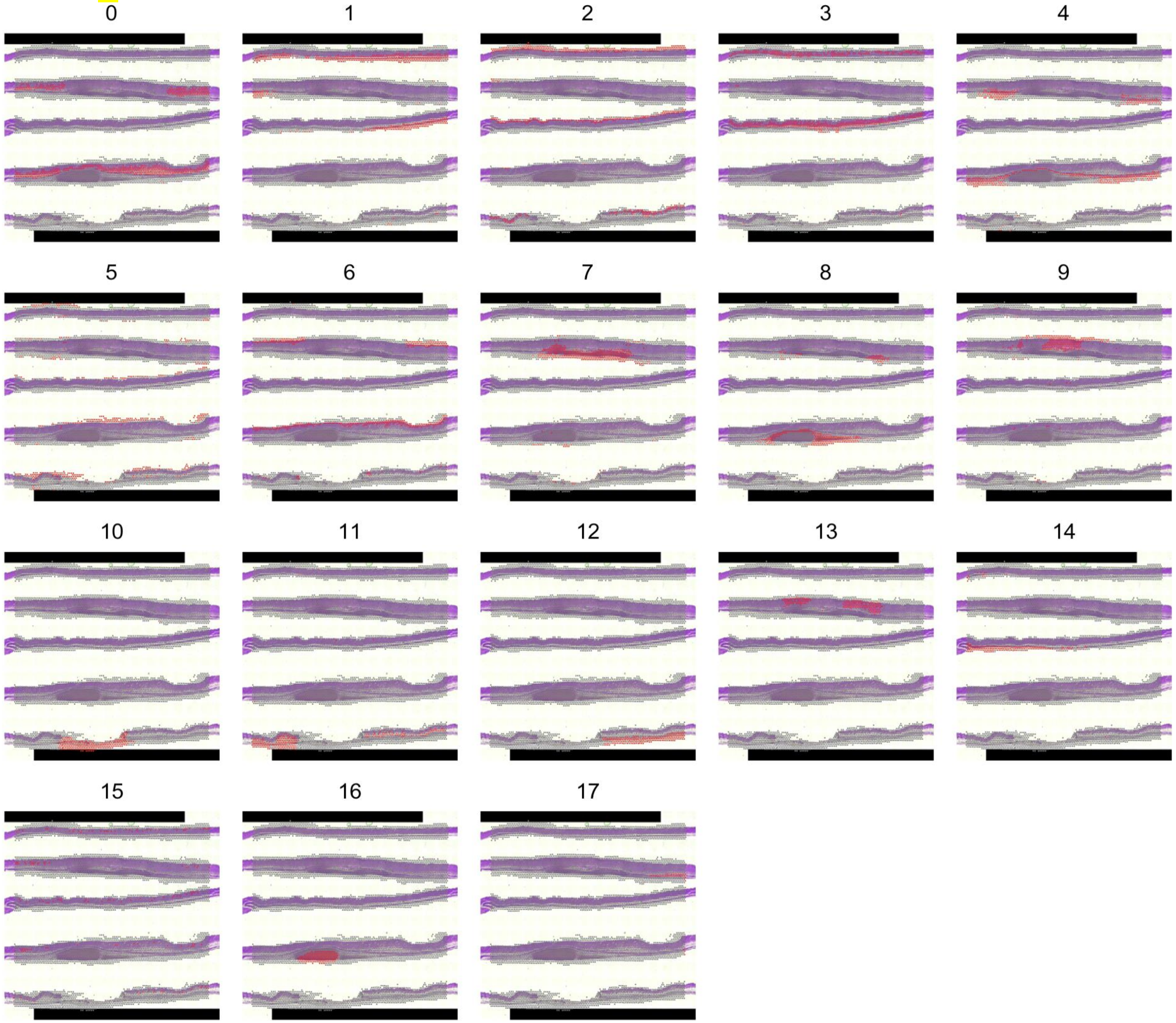

Supplemental Figure 10

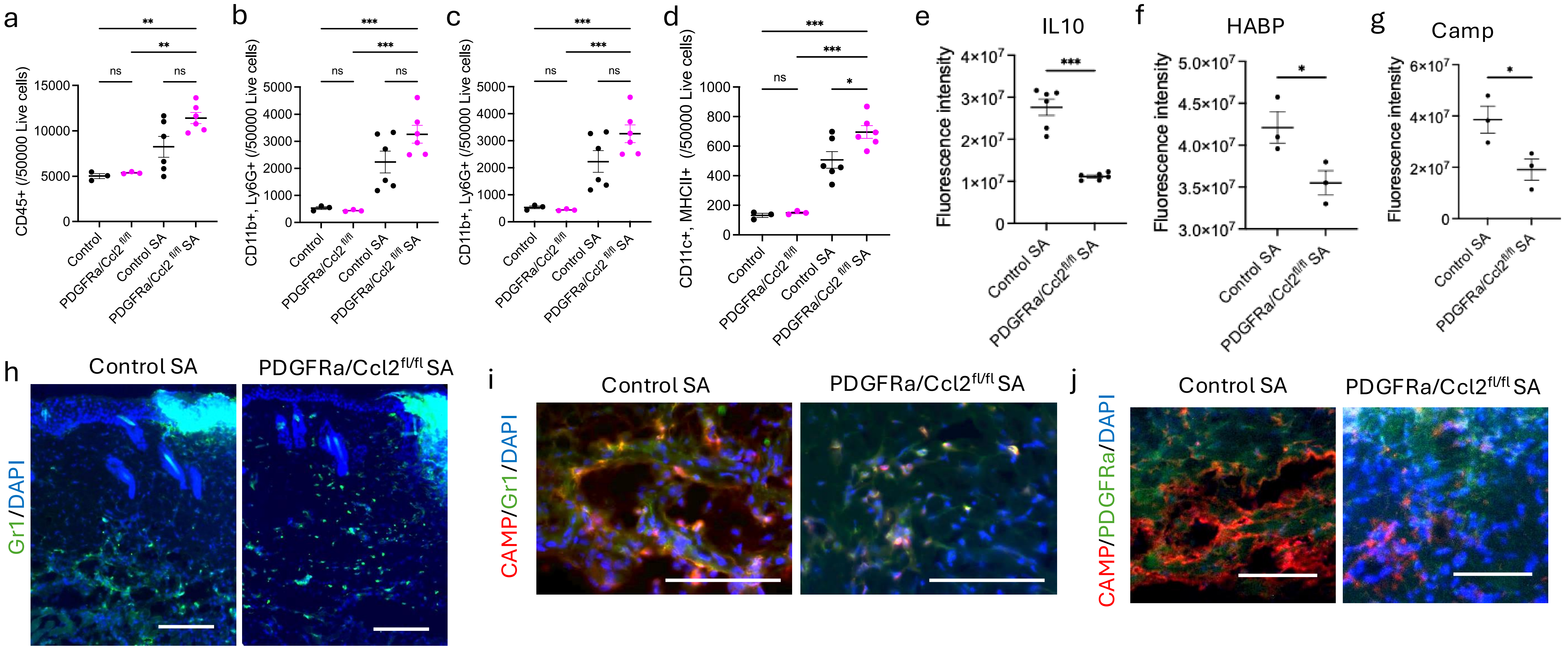

Supplemental Figure 11

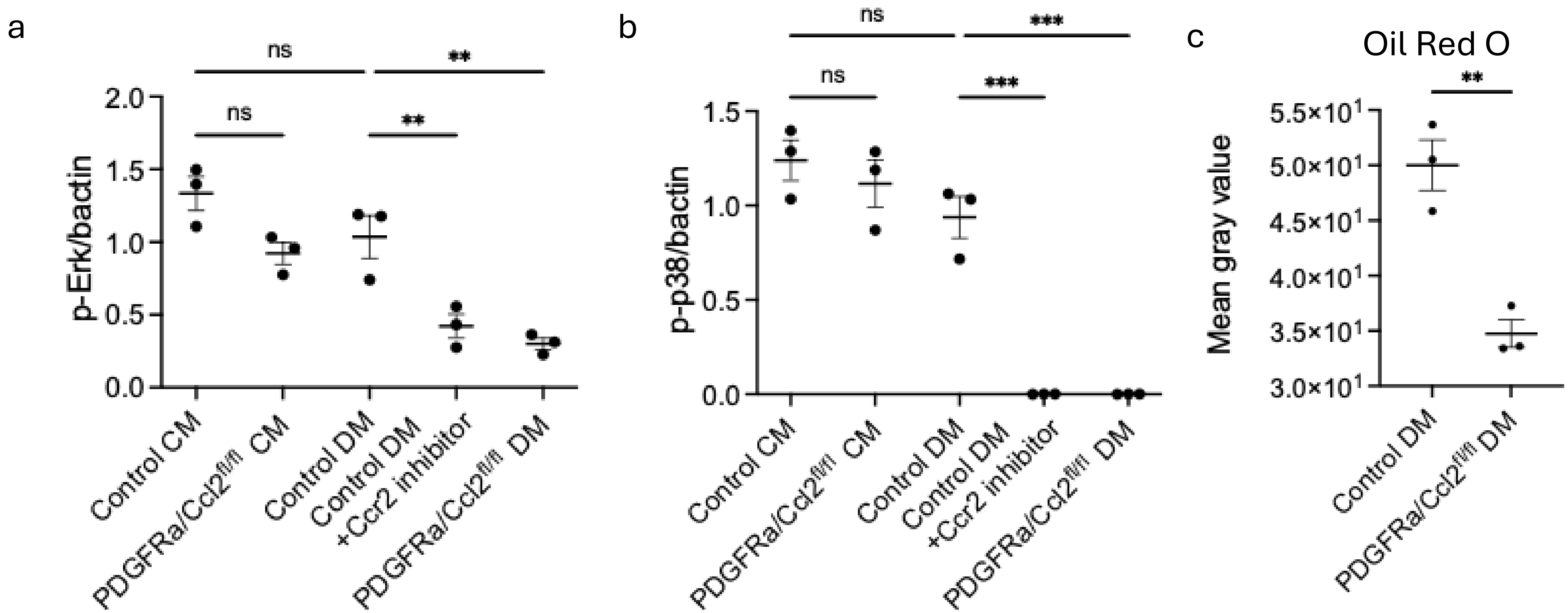

Supplemental Figure 12

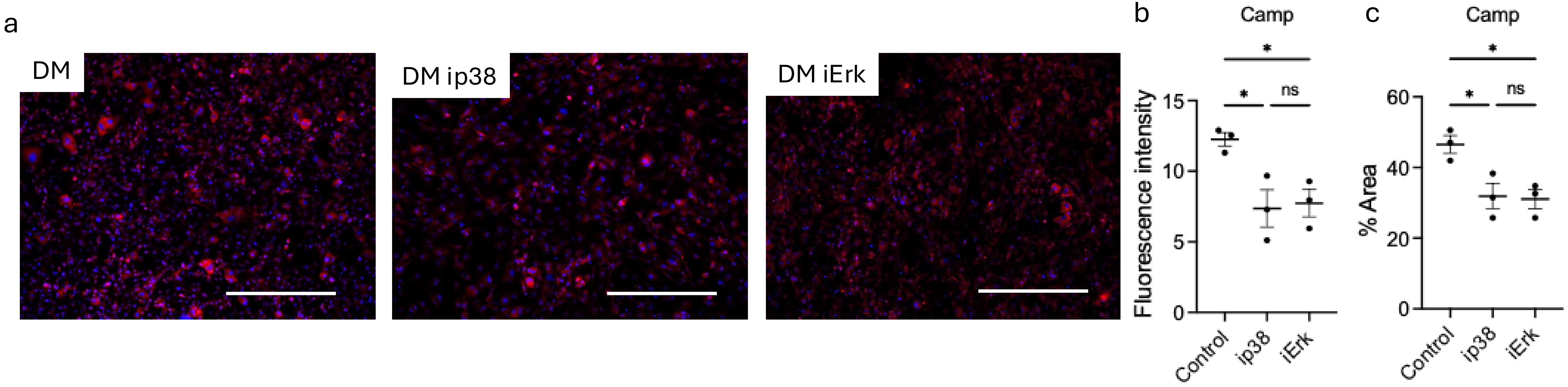

Supplemental Figure 13

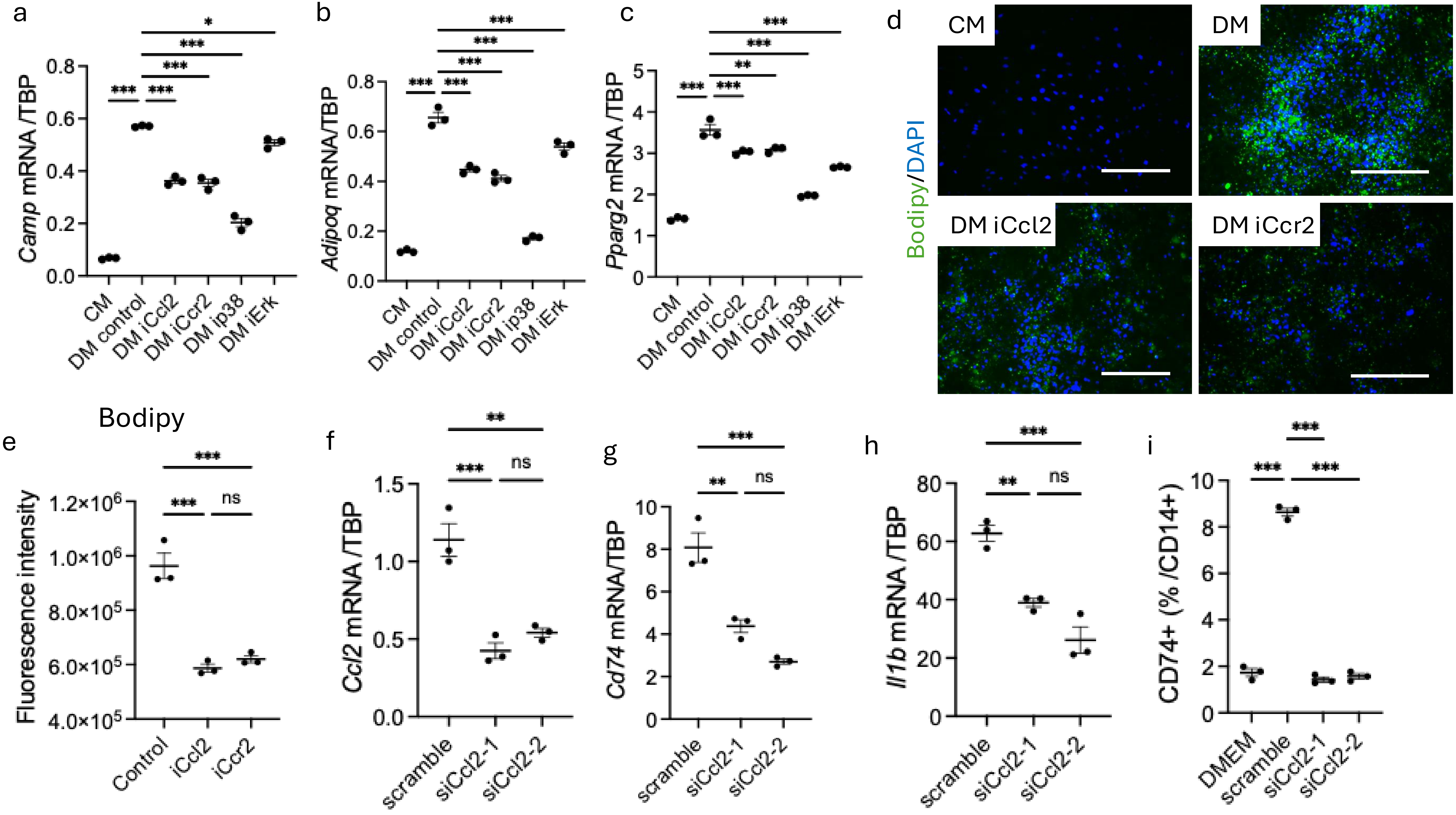

Supplement: Supplementary file 2 — supplemental figures [file 41423_2026_1442_MOESM2_ESM.pdf]
